# Spatial transcriptomics reveals expression gradients in developing wheat inflorescences at cellular resolution

**DOI:** 10.1093/plcell/koaf282

**Published:** 2025-12-13

**Authors:** Katie A Long, Ashleigh Lister, Maximillian R W Jones, Nikolai M Adamski, Rob E Ellis, Carole Chedid, Sophie J Carpenter, Xuemei Liu, Anna E Backhaus, Andrew Goldson, Vanda Knitlhoffer, Yuanrong Pei, Martin Vickers, Burkhard Steuernagel, Gemy G Kaithakottil, Jun Xiao, Wilfried Haerty, Iain C Macaulay, Cristóbal Uauy

**Affiliations:** John Innes Centre, Norwich Research Park, Norwich NR4 7UH, United Kingdom; Earlham Institute, Norwich Research Park, Norwich NR4 7UZ, United Kingdom; John Innes Centre, Norwich Research Park, Norwich NR4 7UH, United Kingdom; John Innes Centre, Norwich Research Park, Norwich NR4 7UH, United Kingdom; John Innes Centre, Norwich Research Park, Norwich NR4 7UH, United Kingdom; Vizgen Inc., 61 Moulton Street, Cambridge, MA 02138, United States; John Innes Centre, Norwich Research Park, Norwich NR4 7UH, United Kingdom; Institute of Genetics and Developmental Biology, Chinese Academy of Sciences, Beijing 100101, China; John Innes Centre, Norwich Research Park, Norwich NR4 7UH, United Kingdom; ICARDA, Biodiversity and Integrated Gene Management, Rabat Institutes, PO Box 6299, Rabat, Morocco; Earlham Institute, Norwich Research Park, Norwich NR4 7UZ, United Kingdom; Earlham Institute, Norwich Research Park, Norwich NR4 7UZ, United Kingdom; John Innes Centre, Norwich Research Park, Norwich NR4 7UH, United Kingdom; John Innes Centre, Norwich Research Park, Norwich NR4 7UH, United Kingdom; John Innes Centre, Norwich Research Park, Norwich NR4 7UH, United Kingdom; Earlham Institute, Norwich Research Park, Norwich NR4 7UZ, United Kingdom; Institute of Genetics and Developmental Biology, Chinese Academy of Sciences, Beijing 100101, China; Earlham Institute, Norwich Research Park, Norwich NR4 7UZ, United Kingdom; Earlham Institute, Norwich Research Park, Norwich NR4 7UZ, United Kingdom; John Innes Centre, Norwich Research Park, Norwich NR4 7UH, United Kingdom

## Abstract

The diversity of plant inflorescence architecture is specified by gene expression patterns. In wheat (*Triticum aestivum*), the lanceolate-shaped inflorescence (spike) is defined by rudimentary spikelets at the base, which form as a result of delayed spikelet and floral development compared with central spikelets. While previous studies identified gene expression differences between central and basal inflorescence sections, gene expression patterns along the apical-basal axis remain poorly resolved due to bulk tissue-level techniques. Here, we optimize Multiplexed Error Robust Fluorescence In Situ Hybridization, a spatial transcriptomics technique, in wheat inflorescence tissue, enabling transcript localization for 200 genes to cellular resolution across 4 stages of development. Cell segmentation and clustering of 50,000 cells identified 18 expression domains and their enriched genes, revealing the spatio-temporal organization of spikelet and floral development, and characterizing tissue-level gene markers. Using these domain- and cell-level maps, we characterize expression patterns of genes differentially expressed across the apical-basal axis. We identify distinct, spatially coordinated expression patterns distinguishing axillary meristems and their subtending leaf ridges across the apical-basal axis before visible spikelet formation, highlighting factors patterning meristem identity and transition. To support the broader research community, all raw and processed data are publicly available, including through an interactive WebAtlas interface (www.wheat-spatial.com).

## Teaser

High-resolution spatial transcriptomics reveals coordinated gene expression patterns that define spikelet and floral development in wheat.

## Introduction

Nature exhibits a stunning diversity of vegetative and flowering structures contributing to plant fitness. Characterizing regulatory genes and their spatiotemporal expression is essential to understanding tissue patterning, as cell fate is guided by positional cues within developing tissues (van den Berg et al. 1995; Kidner et al. 2000; Costa and Shaw 2006). Grass morphology is patterned through phytomers, a basic unit consisting of an internode, leaf, and axillary meristem (AM; Briske 1991; Moore and Moser 1995). During vegetative growth, the shoot apical meristem (SAM) initiates phytomers, with outgrowth of lateral leaf primordia while AMs mostly remain dormant, with the exception of tiller formation (Pautler et al. 2013). This developmental trajectory shifts as the SAM transitions to an inflorescence meristem (IM). Phytomer initiation continues, but leaf (bract) outgrowth is suppressed, and AMs pattern reproductive growth (Whipple et al. 2010; Kellogg 2022).

Grass inflorescences share the basic organization of flowers within spikelets (Clifford 1987; Clayton 1990), but beyond this, inflorescence architecture varies widely due to differences in phyllotaxy and branching (Bartlett and Thompson 2014). As the inflorescence develops, meristems acquire distinct identities that determine the type of primordia they produce. Lateral AMs, initiated on the flanks of the IM, transition through 3 general identities: branch meristem (BM), spikelet meristem (SM), and floral meristem (FM) (Tanaka et al. 2013; Bartlett and Thompson 2014). In panicle-type inflorescences such as rice (*Oryza sativa*), AMs first adopt BM identity, forming primary and secondary branches that generate additional meristems along their flanks (Ikeda et al. 2019). In contrast, in spike-type inflorescences such as those of wheat (*Triticum aestivum*) and barley (*Hordeum vulgare*), AMs transition directly to SM identity without branching. Each SM gives rise to a spikelet, which in turn will produce one or many FM, each producing a single floret (Koppolu and Schnurbusch 2019; Koppolu et al. 2022). It is the precise coordination of meristem identity transitions across space and time that shapes the final structure of the inflorescence.

Early during wheat inflorescence development, in the so-called double ridge stage, the IM initiates pairs of ridges. Each double ridge is composed of a suppressed bract, referred to as the leaf ridge (LR), and an AM, referred to as the spikelet ridge (SR). As these ridge pairs are initiated acropetally, a developmental gradient is established along the nascent inflorescence, with basal phytomers being the oldest (Bonnett 1966; Waddington et al. 1983). However, the timing of each AM's meristem identity transitions (AM > SM > FM) does not follow this age gradient. Instead, central AMs are the first observed to form to glumes (the first indication of SM) and lemma primordia (LP) (indication of FM)—while basal AMs, despite being older, are delayed (Bonnett 1966). This delay persists throughout inflorescence patterning, resulting in basal spikelets forming immature floral structures that fail to produce grains (Backhaus et al. 2022, 2023). These findings highlight the composite nature of the wheat spike, where the interplay between age and positioning coordinates meristematic progression along the apical–basal axis, giving rise to a complex structure.

Given this spatial complexity, the key transcriptional regulators determining the developmental progression/suppression of AMs across the apical-basal axis in wheat remain largely uncharacterized. Previous use of bulk RNA-sequencing of whole inflorescence tissues has obscured spatial variation in gene expression. To address this limitation, semi-spatial transcriptomic techniques have been applied to grass inflorescences, including manual microdissection (Backhaus et al. 2022) and laser capture microdissection (Harrop et al. 2016; Thiel et al. 2021). Low-input RNA-seq of apical, central and basal sections of microdissected wheat spikes revealed large differences in gene expression profiles among them. For example, the MADS-box transcription factor *VEGETATIVE TO REPRODUCTIVE TRANSITION 2 (VRT2)* exhibited its highest expression in basal sections, with decreasing levels toward the apex, in a proposed gradient along the inflorescence (Backhaus et al. 2022). Increased expression of *VRT2* led to a subtle delay in basal spikelet development and elongated organs within the spikelet (glumes and lemmas; Adamski et al. 2021; Backhaus et al. 2022). Given the high levels of differential expression observed across the spike in this experiment, we hypothesized that other genetic factors contribute to apical-basal axis patterning that warrant further investigation. However, semi-spatial resolution experiments leave precise gene expression patterns undefined.

Recently developed spatial transcriptomic approaches enable the quantification of gene expression within the spatial context of tissues and cells (Giacomello 2021; Moses and Pachter 2022). In plants, early applications used single-molecule fluorescence in situ hybridization, which improved upon traditional in situ methods by localizing single RNA molecules to sub-cellular resolution (Duncan et al. 2016). More recent techniques can detect a larger set of genes and fall broadly into 2 categories: sequencing-based and imaging-based (reviewed in Nobori 2025), with both being applied recently to inflorescence tissues (Giacomello et al. 2017; Wang et al. 2024; Demesa-Arevalo et al. 2025; Liu et al. 2025; Xu et al. 2025). Sequencing-based approaches allow for unbiased transcriptome-wide studies, however, in practice, these techniques are limited in the capture of low-abundance transcripts and often compromise on cellular resolution. Imaging-based methods target a predefined set of genes, limiting and biasing the genes detected per experiment, but allow for precise spatial and cellular resolution.

Here, we adapt the imaging-based Multiplexed Error Robust Fluorescence In Situ Hybridization (MERFISH; Chen et al. 2015; Moffitt et al. 2016) protocol to map the spatial expression of 200 genes along the apical-basal axis of the developing wheat spike to cellular resolution. Cell segmentation and clustering across 4 timepoints identified 18 expression domains (EDs) and their enriched gene markers, offering detailed insights into gene expression at tissue and cellular levels. We uncovered detailed spatial and temporal organization in the developing wheat spike, including coordinated transcriptional gradients that distinguish and define leaf and SRs along the apical-basal axis, prior to central meristem outgrowth. To support the broader research community, we developed an open-access WebAtlas interface (Li et al. 2025; www.wheat-spatial.com) enabling visualization of all measured genes and EDs, and a detailed protocol on the preparation of plant samples for spatial transcriptomic work (doi.org/10.17504/protocols.io.rm7vzqwb4vx1/v1). This work highlights the potential of spatial transcriptomics time-series to investigate gene expression patterns to single cell resolution while retaining the tissue morphology and spatial context of each cell in planta.

## Results

### MERFISH of wheat inflorescence resolves gene expression to cellular resolution across 4 developmental timepoints

**Figure 1:**
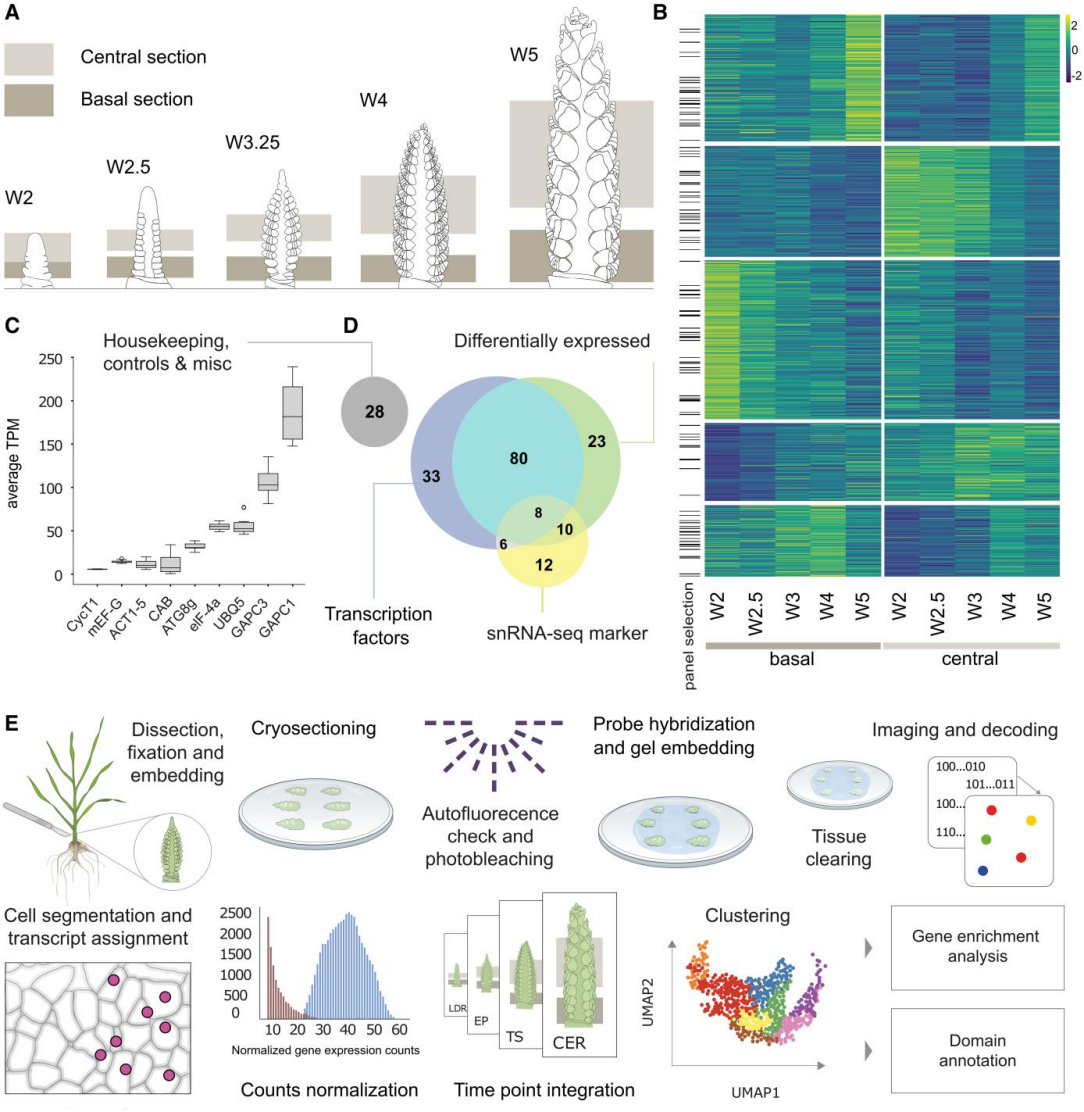
Microdissection of wheat inflorescence reveals differentially expressed genes used to build 200-gene panel in MERFISH workflow. **A)** Schematic illustrating the central and basal spike regions sampled for pooled-tissue RNA-sequencing across 5 developmental stages (W2, W2.5, W3.25, W4, W5; not to scale). **B)** 12,384 genes were differentially expressed between central and basal regions of wheat inflorescence microdissections. The 121 genes selected for inclusion in the MERFISH panel are indicated by black bars. **C)** Average expression (TPM) values for 9 housekeeping genes in microdissected RNA-seq dataset (timepoints W2 to W5), incorporated into MERFISH panel. **(D)** Composition of 200-gene MERFISH panel, including differentially expressed genes selected from microdissection dataset, single-nuclei RNA-seq markers (Liu et al. 2025), additional transcription factors, and housekeeping/control genes. **E)** Workflow of the MERFISH workflow for plant tissues, including sample preparation, imaging, and data analysis.

To characterize gene expression along the apical–basal axis, we first selected genes for spatial profiling. Initial gene selection was informed by a microdissection RNA-seq dataset (Backhaus et al. 2022), however, given its limited developmental range and high variability, we conducted a more extensive analysis across spike development. We generated RNA-seq from central and basal spike sections across 5 development stages (Fig. 1A and B); early and late double ridge (EDR, LDR; Waddington stage W2, W2.5; respectively), LP (W3.25), terminal spikelet (TS; W4), and carpel extension (CE; W5) (Waddington et al. 1983; Kirby and Appleyard 1984). Individual samples expressed, on average, 49,387 high-confidence genes, with 55,346 unique genes expressed across all samples. We identified 12,384 genes with significant differential expression between central and basal sections over time (Fig. 1B; see Materials and methods), consistent with distinct spatial profiles along the apical-basal axis.

The MERFISH workflow begins with curation of a gene panel to guide transcript-specific probe design. We assembled a 200-gene panel investigating wheat inflorescence development, spanning 4 categories: (i) genes differentially expressed along the apical–basal axis, (ii) genes previously annotated as related to cereal inflorescence development, (iii) cell type marker genes identified from a single-nucleus RNA-seq study (Liu et al. 2025), and (iv) housekeeping and control genes. Of these, 121 genes were differentially expressed across the apical-basal axis of the inflorescence in our RNA-seq microdissection dataset. For most genes, including those characterized as “inflorescence development” genes, selection was guided by prior genetic characterization in wheat or orthologs from *O. sativa*, *Zea mays*, and *H. vulgare*. To this set of 172 inflorescence-related genes we included an additional 28 housekeeping, control, and miscellaneous genes for a final panel of 200 genes (Fig. 1C and D; Table S1). An additional 100 grain development genes were included for a parallel project, resulting in a 300-probe set that was subsequently synthesized and used for the Vizgen MERSCOPE workflow (Fig. 1E).

Developing wheat spikes were dissected at 4 stages (Fig. S1; W2.5, W3.25, W4, W5; Waddington et al. 1983), embedded in optimal cutting temperature (OCT) compound, flash frozen, and cryo-sectioned (Fig. S2). Each OCT block included 5 to 36 spikes (depending on stage) from 2 near-isogenic lines: 1 carrying the wildtype *VRT-A2a* allele (*P1^WT^*) and the other the misexpression *VRT-A2b* allele (*P1^POL^*) from *Triticum turgidum* ssp. *polonicum* (Adamski et al. 2021). The detailed workflow of plant tissue embedding and sectioning is provided as a separate protocol (doi.org/10.17504/protocols.io.rm7vzqwb4vx1/v1). To accommodate high auto-fluorescence in plant tissues, we performed autofluorescence checks and increased photobleaching times as required, and increased time submersed in tissue clearing solutions (see Materials and methods). We imaged across 4 experimental runs in total. The size of raw imaging data generated from each experimental run ranged from 460 GB to 1.52 TB.

For downstream analysis, we selected 2 representative samples at each timepoint (one per genotype) for a total of 8 samples, selecting for sectioning angle, tissue adherence, and high transcript capture (Fig. S2). Cell segmentation (Fig. S3; Pachitariu and Stringer 2022) and transcript assignment (Fig. S4; Wiggin and Yu 2024) yielded a cell-by-gene matrix detailing transcript counts per cell for each gene (see Materials and methods). After filtering the dataset to remove segmentation artifacts (non-cellular objects) and low-quality cells with few detected transcripts (see Materials and methods, Fig. S5), we assessed sample quality based on total transcript counts and number of genes detected per cell. Total cells captured per sample range from 1,569 (W2.5, *P1^POL^*) to 15,185 (W5, *P1^WT^*), reflecting the change in inflorescence size across the 4 developmental stages (∼1.2 to 4 mm in length; Kirby and Appleyard 1984). Across all samples, the average transcript counts per cell ranged between 77.9 to 152.4 counts, while average gene counts per cell ranged from 29.6 to 42.1. We observed gene expression patterns at cellular resolution, including transcripts localized to a single cell layer of the epidermis, consistent with MERFISH providing spatial expression data at cellular resolution (Fig. S4).

### MERFISH quality control and verification

We next implemented quality control (QC) measures to evaluate the performance of MERFISH in plants. In MERFISH, each transcript is identified through a unique barcode. To assess the percentage of errors in transcript detection, the MERSCOPE platform detects the presence of 15 “blank” barcodes not included in the experimental library (Chen et al. 2015). These blank barcodes were rarely detected, representing <0.28% of total detections per cell on average (range by sample: 0.24% to 0.34%). Due to the hybridization-based nature of MERFISH and the ∼98% sequence identity among wheat homoeologs, we expected cross-hybridization between homoeologous transcripts. To test this, we included a probe set targeting a B-genome transcript in a triad with little to no B-genome expression. Despite being designed against the B genome copy, the probe detected an average of 0.20 transcripts per cell—10-fold higher than blank controls—indicating it captured A and D homoeologs and a lack of homoeolog specificity, an important consideration for polyploids (Fig. S6).

To further validate our results, we generated in silico “sections” corresponding to physical microdissections and compared their normalized expression data with microdissection bulk RNA-seq. Correlations in basal and central sections across 8 samples and 4 time points yielded an average Spearman's correlation coefficient of 0.66 (range 0.62 to 0.73; Fig. S7), supporting consistency between approaches. Additionally, we compared MERFISH data with published in situ hybridization studies in inflorescence tissues. Given the limited number of studies in wheat, we broadened the comparison to include other cereals and observed broadly consistent expression patterns. (Fig. S8). These results demonstrate that our MERFISH data exhibits high QC metrics, is non-homoeolog specific, and is consistent with established gene expression patterns, confirming the technique's robustness in plant tissues.

### Leiden clustering defines 18 EDs across developmental time

**Figure 2:**
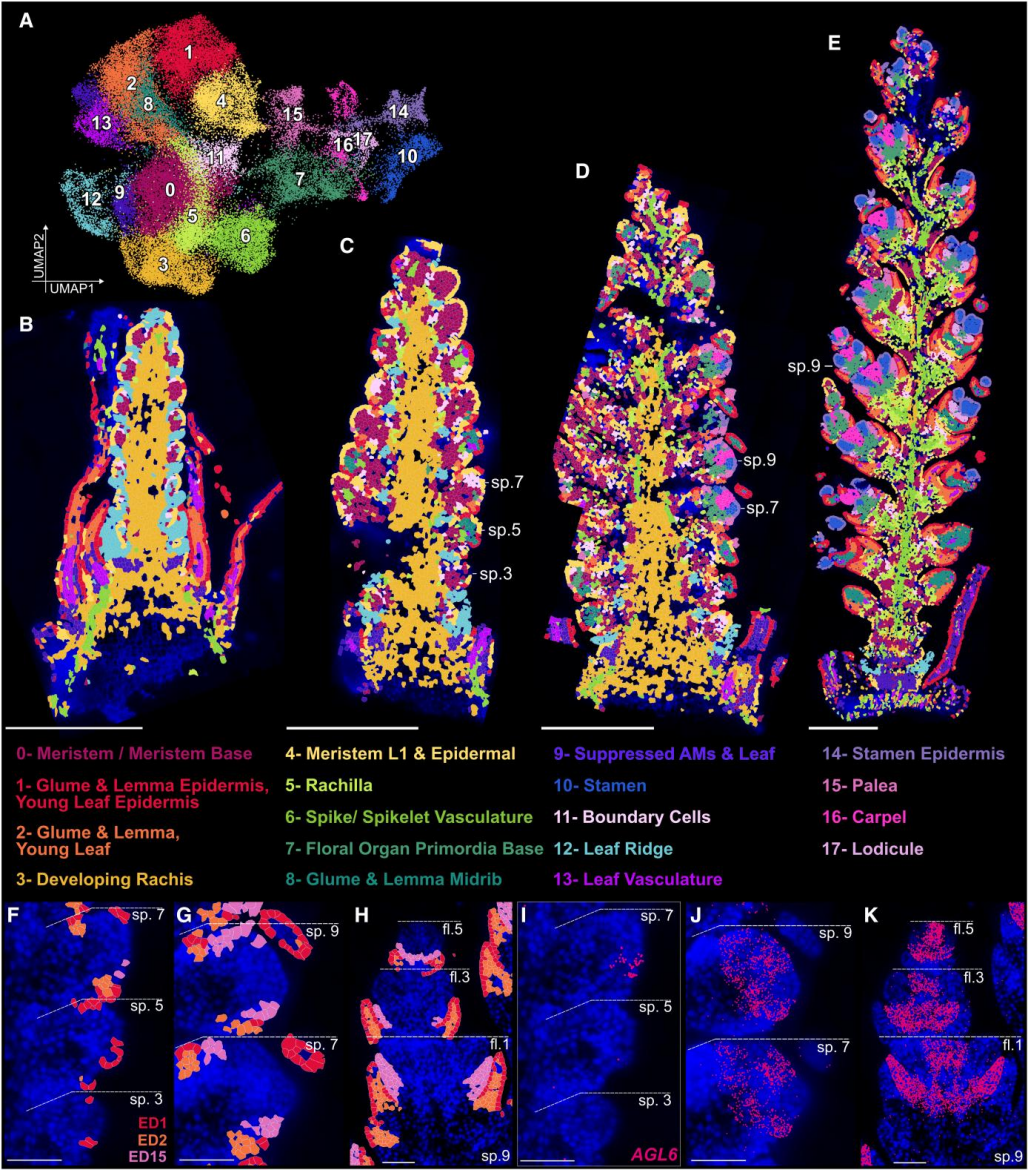
Leiden clustering identifies 18 EDs mapped over 4 developmental stages. **A)** UMAP projection of 50,731 cells from 8 samples (4 developmental stages, 2 genotypes), and ED assignment. **B** to **E)** Spatial maps of Leiden clustering across time points **(B)** W2.5, **(C)** W3.25, **(D)** W4, and (e) W5 using Squidpy (v1.4.1), Scanpy (v 1.10.0), and Scanorama (v1.7.4); scale bar = 500 *µ*m. Spikelet numbers labeled, as referenced in subsequent domain and transcript maps (sp. = spikelet). **F** to **H)** Spatial plots of cell segmentation and assigned EDs 1, 2, and 15 in **(F)** W3.25 spikelets 3,5,7 (g) W4 spikelets 7,9 (h) W5 spikelet 9 (florets = fl; 1,3,5). **(I** to **K)** Expression of *AGL6* in same tissues as **(F** to **H)**, respectively. Blue = DAPI staining; Scale bar = 100 *µ*m.

Transcript counts were normalized for samples individually (Fig. S5), followed by sample integration to group 50,731 total cells across the 4 developmental stages and 2 genotypes, and subsequent unsupervised clustering (Wolf et al. 2018; Palla et al. 2022). This pipeline yielded 18 distinct ED (Fig. 2A), which were visualized as spatially resolved maps (Fig. 2b to E). The total number of clusters per sample increases through developmental time, with WT samples W2.5, W3.25, W4, W5, summarized by 11, 12, 17, and 18 domains, respectively (removing clusters representing <0.5% of cells per sample, see Materials and methods). We observed EDs remained consistent in their patterning across biological replicates (Fig. S9), in addition to across time points. For example, ED3 maps consistently to the developing rachis in W2.5, W3.25, and W4 in both *P1^WT^* and *P1^POL^*, followed by the relative decrease in ED3 and increase in ED6 (vasculature) of the W4 spike. In some cases, we observed the clustering of 2 separate tissues into one domain. Glumes and lemmas are clustered in domain (ED1 + 2) with young leaf tissue in stage W2.5, potentially highlighting their transcriptional similarities and consistent with the classification of glumes and lemmas as homologous to leaf-like bracts (Kellogg 2000; Patterson et al. 2023).

Notably, 6 domains were primarily detected at stages W4 and W5 (ED16, 14, 7, 15, 10, 17). which we identified as floral tissues (Fig. 2D and E) based on their spatial distribution and organization within the inflorescence. We hypothesized that concatenating samples across time would reveal floral cells in their earliest stages of emergence. For example, in W3.25 spikes, we identified only 3 ED15 cells (Fig. 2F), a predominant domain in more mature spikes (Fig. 2G) and which later localizes to paleae in W5 samples (Fig. 2H). At W3.25, these 3 ED15 cells were positioned just above the LP (ED1, 2), a spatial arrangement consistent across stages (Fig. 2F to H). In all ED15 cells, we observe the expression of *AGAMOUS-LIKE6 (AGL6)*, a key regulator of palea identity in wheat (Fig. 2I to K; Kong et al. 2022). This indicates that ED15 at W3.25 most likely represents the first cells with palea identity in the developing spike.

### Gene expression analysis defines identity of EDs

Beyond cell clustering, all generated EDs require tissue specific annotations in order to gain biological insights. To do so, we annotated the 18 domains by combining knowledge of anatomical features, with the identification of domain-enriched genes (Table S2). Domain-enriched genes are defined as those exhibiting significantly elevated expression in a domain relative to others, thereby providing transcriptomic signatures that distinguish each ED. We characterized 54 genes from our panel to show domain specific enrichment. A majority of these markers (57.4%) were enriched in multiple EDs, with 23 genes representing only one ED (Table S2). Ten genes were enriched in >3 clusters and included markers of vegetative tissues and bracts (*YABBY7*), outer cell layer (orthologs to *OsONI1*, *OsROC3t*, *OsROC7t*), meristematic and ground tissue (*KNOX5*), and floral tissues (*AP3*, *AGL6*, *SEP3-1*, *SEP3-2*).

We identified 8 distinct EDs corresponding to floral tissues and their subtending bract tissues (glume + lemma) in wheat, defined by the enrichment of 29 genes, including 10 MADS-MIKC transcription factors. According to the ABCDE and floral quartet models, floral organ identity is governed by combinatorial tetrameric complexes of these MADS-domain proteins, which regulate downstream targets involved in organ specification (Coen and Meyerowitz 1991; Ma and dePamphilis 2000; Theißen et al. 2016; Bowman and Moyroud 2024). In grasses, floral organs are arranged into 4 whorls, with identity determined by specific combinations of MADS-box proteins: A- and E-class factors specify the palea (whorl 1); A-, B-, and E-class define the lodicules (whorl 2); B-, C-, and E-class specify stamens (whorl 3); C- and E-class determine carpels (whorl 4), and D-class genes are primarily involved in ovule development (Whipple and Schmidt 2006; Thompson and Hake 2009; Murai 2013).

**Figure 3:**
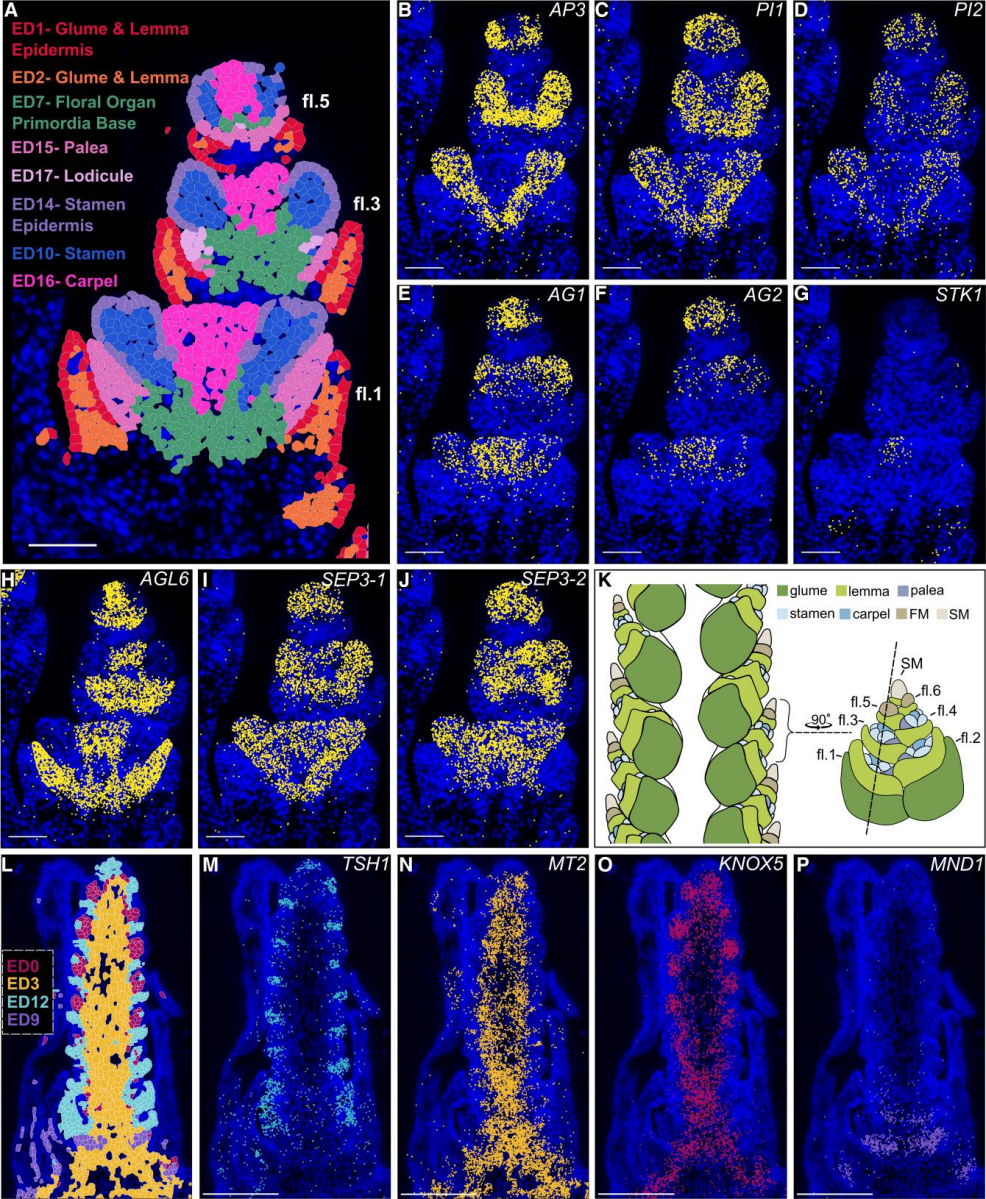
Enrichment analysis highlights domain-specific gene expression profiles. **A)** Eight EDs in W5 *P1^WT^* florets mark floral organ identity (ED1 + 2 = glume/lemma, ED7 = floral organ primordia base, ED15 = palea, ED17 = lodicule, ED14 + 10 = stamen, ED16 = carpel) fl = floret. **B** to **J)** Spatial transcript localization in W5 florets, **(B)**  *AP3*, **(C)**  *PI1*, **(D)**  *PI2*, **(E)**  *AG1*, **(F)**  *AG2*, **(G)**  *STK1*, **(H)**  *AGL6*, **(I)**  *SEP3-1*, **(J)**  *SEP3-2.* Scale bar = 100 *µ*m. The image in panel **(H)** is the same as that in Figs. 2K and S8d. The image in panel (o) is the same as Fig. S11e. The image in (p) is the same as Fig. S8k. The images in panels (b to j) are derived from imaging the same cryosection; additional images from this cryosection are shown in Fig. S10b to l. **K)** Diagrammatic representation of wheat floral anatomy and cryosection angle (black dashed line). This sectioning angle is representative of the cryosection displayed in panel (a). fl, floret; SM, spikelet meristem; FM, floral meristem. l) Spatial map of 4 EDs in W2.5 spikes, highlighting domains enriched with transcripts in subsequent panels. ED0, meristem and meristem base; ED3, developing rachis; ED9, suppressed axillary meristem and leaves; ED12, leaf ridge. m to p), (m) *TSH1*, (n) *MT2,* (o) *KNOX5*, and (p) *MND1.* Scale bar = 250 *µ*m. Blue stain = DAPI. The images in panels (l to p) are derived from imaging the same cryosection; additional images from this cryosection are shown in Figs. 5F, G and S11a to k.

The spatial co-expression of ABCDE genes provided an ideal benchmark to evaluate whether MERFISH can resolve combinatorial gene expression patterns that underlie cell identity. Based on their positioning within the florets, we identified lemma (ED1 + 2) palea (ED15), lodicules (ED17), stamen (ED10 + 14), and carpel (ED16; Fig. 3A). Consistent with the ABCDE model, B-, C-, and D-class genes showed spatially restricted expression (Fig. 3B to J), helping to differentiate floral tissues into EDs. For example, both lodicules and stamens are enriched in B-class genes *APELATA3* (*AP3*, ortholog to *OsMADS16*), *PISTILLATA-1* (*PI1*, *OsMADS4*), and *PISTILLATA-2* (*PI2*, *OsMADS2*). However, stamens are differentiated by the expression of C-class genes *AGAMOUS-1* (*AG1*, *OsMADS58*; Fig. 3E), absent from lodicules. Similarly, the D-class gene *SEEDSTICK1 (STK1)* is restricted to the carpel domain (Fig. 3G). In addition to proposed members of the ABCDE model, lodicules, palea, glumes, and lemma are enriched in *YABBY7*, an ortholog to a marker of lateral organs in rice (*OsTOB2*, Tanaka et al. 2017; Fig. S10). Despite this shared expression pattern, glumes and lemmas are clustered together in ED1 + 2, and the distinction of palea and lodicules into their own domains comes from the expression of E-class *AGL6* genes. This distinction is consistent with the classification of palea and lodicules as the first 2 whorls in floral tissues, as opposed to the bract-like (non-floral) identity of glumes and lemmas (Kellogg 2000; Patterson et al. 2023).

While we observe the conservation of expression patterns in B-, C-, and D- class genes, we note the divergence in the expression of E-class genes compared with other monocots. In the ABCDE model, E-class genes are required for the formation of all floral organs (Pelaz et al. 2000; Honma and Goto 2001; Ditta et al. 2004). In the case of the E-class LOFSEP clade, expression of orthologs to *SEPALLATA1* (*SEP1-1*, *SEP1-2*, *SEP1-4*, *SEP1-5*, *SEP1-6*) are mostly absent from floral organ primordia (Fig. S10), the significance of which is further explored in Note S1. However, the E-class genes *SEPALLATA 3-1* (*SEP3-1*), *SEP3-2*, and *AGL6* display overlapping, not fully redundant, enrichment across all floral organs. *AGL6* is expressed in palea, carpels, and lodicules; while *SEP3-1* and *SEP3-2* transcripts are observed in stamens, carpel, and lodicules, an expression pattern that is conserved in a similar spatial transcriptomic study in barley (Demesa-Arevalo et al. 2025).

In early inflorescence development, ED12 is enriched for *TraesCS1A02G418200*, the ortholog of maize (*Z. mays*) *tassel sheath1* (*tsh1*), which is only expressed in ED12 cells (Fig. 3L and M). ED12 is also enriched in transcripts encoding YABBY transcription factors, including *YABBY1*; however, *YABBY1* is also expressed in other lateral organs across development, including leaves (ED13) and lemma/glumes (ED1 + 2). Some genes uniquely mark single domains, such as *METALLOTHIONEIN 2* (*MT2*; Fig. 3L and N), found in the developing rachis (ED3), while others are enriched across multiple domains, such as *KNOTTED1-LIKE HOMEOBOX 5* (*KNOX5*) in the developing rachis (ED3) and meristematic cells (ED0; Fig. 3L and O). We observe *KNOX5* expression is excluded from the tunicate/L1 layer, consistent with observations of its maize ortholog *knotted1* (Jackson et al. 1994). Certain domains encompass multiple tissues: ED9 marks both developing young leaves and suppressed AM below the inflorescence per se, with *MANY NODED DWARF 1* (*MND1*) marking suppressed AMs only (Fig. 3L and P). We infer that the spike-focused probe panel lacked sufficient genes to distinguish these tissues as distinct domains.

### The spatial restriction of *VRT2* and *SEP1-4* is disrupted in the *VRT-A2b* mutant

**Figure 4:**
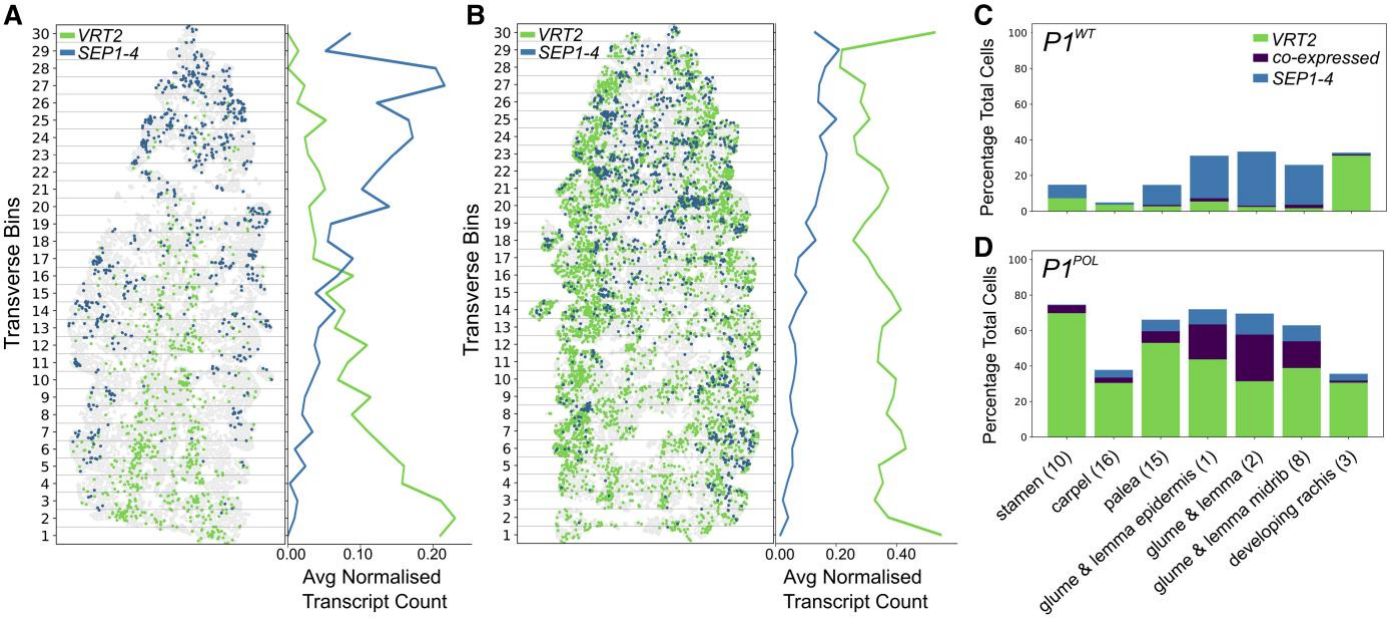
Opposing and spatially restricted gene expression patterns of *VRT2* and *SEP1-4* are disrupted in *P1^POL^*. a and b) Spatial plot of *VRT2* and *SEP1-4* expression in **(A)**  *P1^WT^* and **(B)**  *P1^POL^* W4 spikes, divided into 30 transverse bins along the apical-basal axis, with average normalized expression counts of *VRT2* and *SEP1-4* per transverse bin, normalized with sc.pp.normalize_total and sc.pp.log1p functions (Scanpy v1.10.0). Note difference in *x*-axis scale for (a) and (b). c and d) Proportion of cells expressing either *VRT2, SEP1-4*, or co-expressing both. Calculated as the percentage of total cells per cluster type in **(C)**  *P1^WT^* and **(D)**  *P1^POL^* near-isogenic lines.

We previously identified differences in gene expression between spike sections using microdissection. *VRT2* was most highly expressed in basal sections, with lower expression in the apex, whereas *SEP* MADS-box transcription factors displayed the opposite pattern (Backhaus et al. 2022). *VRT2* is proposed to disrupt SEP–SQUAMOSA complex formation critical for normal spikelet development through a protein competition model (Li et al. 2021), however the co-expression of *VRT2* and *SEP* genes remains uncharacterized. To quantify these profiles, we computationally dissected *P1^WT^* spikes into 30 transverse bins at W4, confirming opposing expression patterns along the apical-basal axis to a finer scale than microdissections. We confirmed the high expression of *VRT2* in the base of the inflorescence. Mean counts per cell of *VRT2* in bins 1 to 10 (basal) is 3.46 × higher than in bins 11 to 30 (central/apical; Fig. 4A). Additionally, MERFISH results revealed the spatial restriction of these 2 genes across the inflorescence. *VRT2* was primarily expressed in developing rachis (ED3) with 32.2% of ED3 cells expressing *VRT2* and showed minimal expression in spikelet tissues such as glumes/lemmas (ED2, 3.3%). In contrast, *SEP1-4* was largely absent from the developing rachis (ED3, 1.4%) but enriched in spikelet tissues including glumes/lemmas (ED2, 31.1%; Fig. 4C). Across the *P1^WT^* spike, only 0.7% of cells co-expressed *VRT2* and *SEP1-4*.

We next asked if MERFISH could quantify the mis-expression of *VRT2* in *P1^POL^*, a near-isogenic line carrying the *VRT-A2b* allele. MERFISH revealed ectopic *VRT2* expression, and disruption of the heightened *VRT2* signal at the base of the inflorescence, whereas the *SEP1-4* expression pattern remained intact (Fig. 4B). This ectopic expression pattern led to increased co-localization of *VRT2* and *SEP1-4*, with 8.2% of cells co-expressing both transcripts along the spike. Co-expression was most pronounced in tissues exhibiting the strongest phenotypic effects in *P1^POL^*, glumes and lemmas, where 26.3% of ED2 cells co-expressed both genes (compared with 1.1% in *P1^WT^*; Fig. 4C and D). These findings establish the spatial restriction of gene expression patterns between *VRT2* and *SEP1-4*, which is disrupted in *VRT-A2b* mutants, and demonstrate the ability of MERFISH to detect tissue-specific changes in gene co-localization in a developmental mutant.

### Transcriptional states differentiate SMs and LRs along the spike

**Figure 5:**
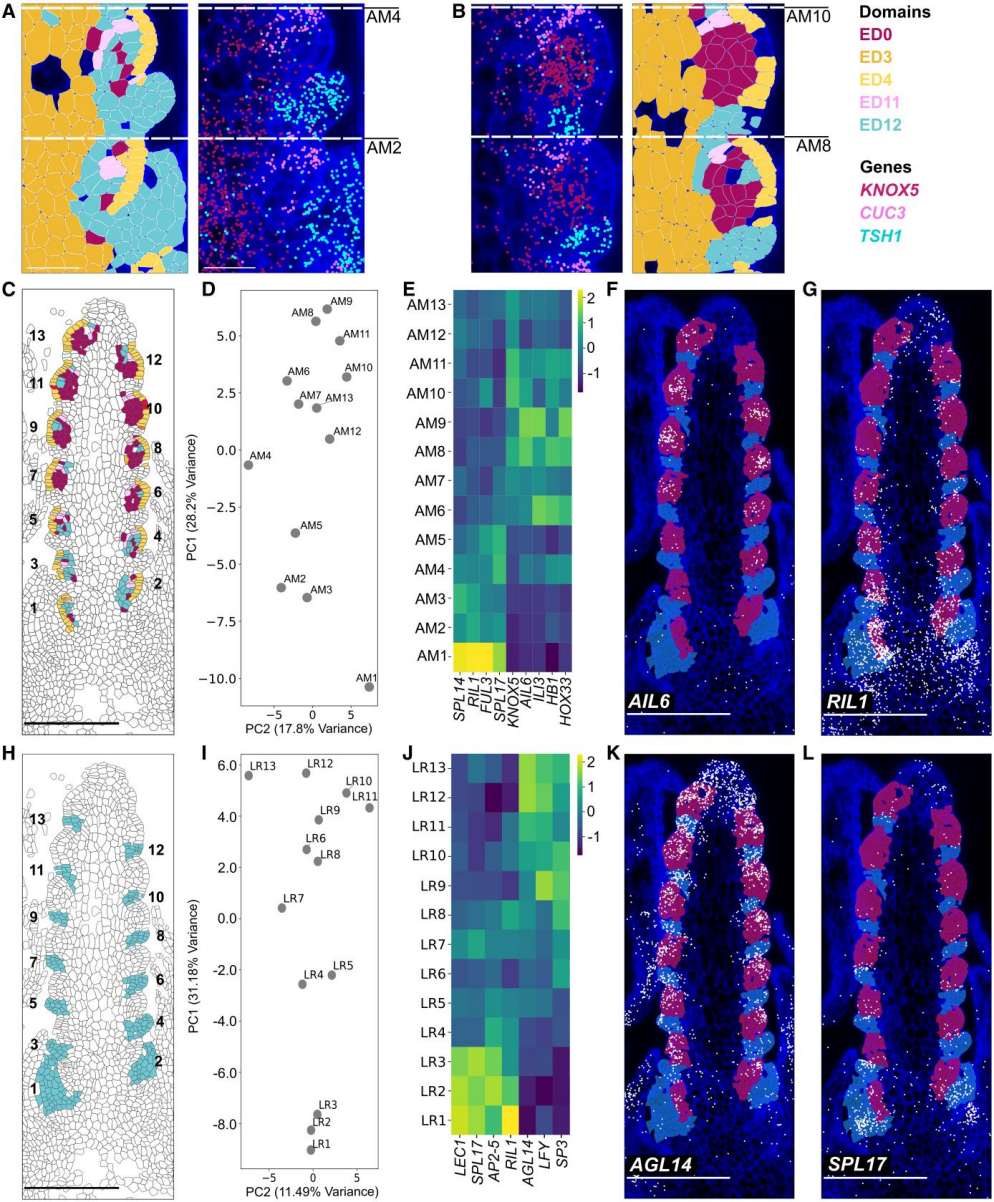
Coordinated and distinct gene expression patterns define LRs and SRs across the apical-basal axis at double ridge stage. **A** and **B)** Basal (2,4) and central (8,10) AMs differ in ED assignment and marker gene expression. *TSH1* and *CUC3* mark the suppressed LR and the adaxial boundary of the SM, respectively. *KNOX5* in layers L2/L3 highlights transcriptional differences between AMs along the apical-basal axis. **C)** EDs define AMs from 1 (basal) to 13 (apical). **D)** PC analysis of averaged transcripts per AM group. **E)** Normalized gene expression (*Z*-score) of select genes with highest/lowest PC1 loadings from analysis in **(D)**. **F** and **G)** Transcript localization in W2.5 spikes of **(F)**  *AIL6* and **(G)**  *RIL1*. Cells in each LR are highlighted in blue, and AMs in pink. **H)** EDs define LRs from 1 (basal) to 13 (apical). **I)** PC analysis of averaged transcripts per LR group. **J)** Normalized gene expression (*Z*-score) of select genes with highest/lowest PC1 loadings from analysis in **(H)**. k and **L)** Transcript localization in W2.5 spikes of **(K)**  *AGL14* and **(l)**  *SPL17.*

The chronological initiation of lateral AMs in the wheat inflorescence does not coincide with their developmental progression. Basal AMs, though first to initiate, lag behind in development compared with central AMs. By the glume primordium stage (W3), central AMs display a visible progression to SM identity, marked by the formation of glumes, while basal AMs remain less developed (Bonnett 1966). We hypothesized that additional gene expression patterns, beyond *VRT2* and *SEP1-4*, may influence these differences before W3. To explore this, we analyzed transcriptional and ED patterns in W2.5 spikes. At this stage, the spike has a relatively simple composition, with 4 domains accounting for 94.8% of the inflorescence cells. By contrast, at W3.25, 8 domains account for a comparable proportion of cells (94.1%). The W2.5 AMs comprise 4 domains: the L1 layer (ED4), meristematic cells in layers L2/L3 (ED0, ED12), and boundary cells (ED11) marking the adaxial boundary. While all AMs exhibit similar L1 (ED4) and boundary (ED11) patterns, basal AMs lack well-defined ED0 regions (Fig. 5A and B). Additionally, while all LRs are represented by one domain (ED12), basal LRs are larger, averaging 32.5 ± 15.8 cells per section (LR1-4), compared with 12.5 ± 1.5 cells in central LRs (LR8-11). These findings support the idea that a wide set of gene expression patterns, contribute to the differentiation of AMs and LRs across the apical-basal axis during or before W2.5.

To quantify differential gene expression patterns within the same tissue type across the inflorescence, we grouped cells from each AM, ordered them longitudinally from basal (1) to apical (13; Fig. 5C), and performed principal component (PC) analysis using a matrix of average counts per cell in each cell grouping (Fig. 5D). PC analyses revealed a strong positive correlation (*ρ* = 0.68, *P* = 0.01) between PC1 and location along the apical-basal axis, with lower PC1 scores associated with basal AMs. (Fig. 5D). Given this relationship, we investigated genes with the highest and lowest PC1 loadings. Genes with lowest PC1 loadings, such as *AINTEGUMENTA-LIKE6* (*AIL6*, *−0.177*), *KNOX5* (−0.175), *HOMEOBOX DOMAIN-1* (*HB1*, *−0.170*), *INCREASED LEAF INCLINATION 3* (*ILI3*, −0.167) are expressed highly in central/apical AMs (Fig. 5E and F and S11). In the case of *KNOX5,* the ortholog to meristematic identity gene maize *knotted1* (Kerstetter et al. 1997), we observed high levels of expression in the meristem corpus of central AMs, which is absent in basal AMs (Fig. 5A and B). Additionally, we observed the wheat ortholog to barley *COMPOSITUM 1* (*HvCOM1/HvTCP24)* was specifically expressed in ED11 cells within AM7,9,10 (Fig. S11). In barley, *COM1* has been shown to inhibit inflorescence branching and thereby promote transition to SM identity (Poursarebani et al. 2020). In contrast, genes with highest PC1 loadings, including *RACHIS-LIKE1* (*RIL1, 0.186*) and *SQUAMOSA-PROMOTER BINDING PROTEIN-LIKE 14* (*SPL14,* 0.180), 2 genes whose orthologs are associated with inflorescence branching in rice (Wang et al. 2015; Ikeda et al. 2019), showed higher expression in basal AMs (Fig. 5E and G).

The equivalent PC analysis on LRs also revealed a correlation between PC1 and position along the apical-basal axis (*ρ* = 0.97, *P* = 2.62e−08; Fig. 5H and I). Genes with the highest PC1 loadings, such as *LEAFY COTYLEDON1* (*LEC1*, 0.179), *APETALA2-5* (*AP2-5*, 0.179), *SPL17* (0.171), and *RIL1* (0.159) were more highly expressed in basal LRs, whereas *AGL14* (−0.171) is more highly expressed in central/apical LRs (Fig. 5J to L). Some genes distinguish between basal and central/apical sections for both LRs and AMs. Ortholog to *SHORT PANICLE 3 (SP3)* is expressed in distinct bands in central/apical regions of both AMs and LRs, while *RIL1* marked both tissues at the base. Notably, *LEC1* was expressed in basal AM1–3 and LR1–3, but from position 4 onward was restricted to AMs (Fig. S11). In contrast, *SPL17* showed LR-specific expression in a position-dependent manner, expressed only in basal LRs (Fig. 5L), a gene whose ortholog in maize *(tassel sheath4)* regulates the control of bract suppression and BM initiation (Xiao et al. 2022). Together, these results provide strong evidence that apical-basal patterning in the developing spike is established before W3 through distinct, tissue-specific gene expression gradients that differentiate AM and LR primordia, and highlight key regulators—such as *RIL1*, *LEC1*, and *SPL17*—that contribute to early axial patterning across multiple cell types. These gradients further suggest that basal AMs exhibit a developmental delay by W2.5, and that this is maintained at subsequent stages (Fig. S12).

## Discussion

### Optimization of MERFISH technology to plant tissues

Spatial transcriptomics is emerging as a powerful tool in plant biology, yet imaging-based approaches like MERFISH face distinct technical challenges when applied to plant tissues. Preparing plant samples requires optimization tailored to tissue type; precise sectioning through complex 3D structures demands both anatomical understanding and technical skill, while the steps required to prepare tissues prior to imaging—such as photobleaching and tissue clearing—vary depending on tissue morphology and composition (Giacomello et al. 2017). Here, we adapted this technique to wheat inflorescences and detail a protocol for tissue preparation from embedding to imaging.

We observed gene expression patterns obtained with MERFISH to be reproducible between independent tissue sections, and consistent with expression patterns observed in single gene in situ hybridization. However, preserving RNA integrity during sampling and sectioning proved particularly challenging, limiting the number of sections available for onward analysis. We observed variability in transcript capture per cell, even among replicate sections from the same developmental stage, which limits direct comparisons of gene expression levels across samples. Moreover, the restricted size of a 200-gene panel constrained transcriptomic coverage. As such, MERFISH has not yet achieved the breadth of coverage possible from snRNA-seq approaches applied to the same tissues (Liu et al. 2025; Xu et al. 2025). Nevertheless, MERFISH enabled simultaneous detection of 200 genes with precise spatial and cellular resolution, representing a major advance over traditional single gene in situ hybridization and microdissection-based transcriptomic techniques.

### MERFISH quantifies gene co-expression key to development

By detecting hundreds of transcripts simultaneously, MERFISH enabled a tissue-specific analysis of gene co-localization. We used this to investigate how a *cis*-regulatory mutation in *VRT2* (*P1^POL^* allele), previously shown to increase *VRT2* expression (Adamski et al. 2021; Backhaus et al. 2022), alters its spatial expression. MERFISH revealed the *P1^POL^* allele drives ectopic *VRT2* expression into spikelet and floral tissues, resulting in elevated co-localization with *SEP1-4*, potentially inhibiting the formation of SEP-SQUAMOSA protein complexes required for spikelet development (Li et al. 2021). In this context, the utility of MERFISH was not in quantifying relative transcript abundance (which can be captured by bulk RNA-seq), but in resolving co-expression networks as spatial domains are reprogrammed in a regulatory mutant. Additionally, while co-expression of *VRT2* and *SEP1-4* could be inferred from snRNA-seq, MERFISH uniquely revealed both the extent of their ectopic co-expression and their spatial distribution, providing mechanistic insight into how *P1^POL^* alters spikelet architecture.

Similarly, MERFISH revealed the co-localization of MADS-MIKC transcription factors in wheat floral tissues, allowing us to connect gene co-expression with floral organ identity. These tissue specific patterns align with phenotypes of homeotic mutants in grasses. For example, B-class genes *AP3*, *PI-1*, and *PI-2* are expressed in lodicules and stamens, consistent with the homoeotic transformation of these organs in rice *ap3* mutants (lodicules to palea-like structures, stamens into carpels (Nagasawa et al. 2003) and in maize *silky1* mutants (Ambrose et al. 2000). Similarly, the expression of C-class *AG1* and *AG2* are expressed in the stamen and carpel, consistent with the rice double mutant (orthologs *osmads3* and *osmads58*), which forms lodicule and carpel-like organs in place of the stamens and carpel (Dreni et al. 2011; Sugiyama et al. 2019). Additionally, *AGL6* distinguishes palea cells (ED15) from glumes and lemmas (ED1 + 2) in clustering analyses, and is enriched in palea, carpel, and lodicules. In tetraploid wheat, *AGL6* double mutants produce lemma-like paleas, lack lodicules, develop ectopic organs between the second and third floral whorls, and display loss of carpel determinacy (Kong et al. 2022).

### EDs trace tissue identity over development

Using domain assignment and gene enrichment, we quantified the transcriptional programmes distinguishing vegetative and inflorescence phytomers in wheat, expanding upon genetic characterization across the grasses. In vegetative tissues, AMs (ED9) located in the axils of leaves (ED1, 2, 13), are enriched in *MND1*, a gene involved in AM suppression in barley (Walla et al. 2020). *MND1* expression remains consistent in AM cells found below the inflorescence at stages W3.25 and W4, suggesting the continued suppression of meristem outgrowth. In contrast, inflorescence phytomers are characterized by the presence of SRs (ED0, 4, 11, 12), which alternate with LRs (ED12). The bract suppression gene *TSH1*, conserved across maize, rice, and barley (Wang et al. 2009; Whipple et al. 2010; Houston et al. 2012), is specifically expressed in LRs, highlighting a shift in lateral organ fate. This transition, from suppression of AMs in vegetative phytomers to suppression of LRs in inflorescence phytomers, reflects a key developmental reprogramming event in the wheat shoot apex at the onset of inflorescence formation.

By integrating data across 4 developmental time points prior to cell clustering, we traced changes to ED composition across the apical–basal axis as the lanceolate shape of the spike emerged. LRs (ED12) were consistently present across all inflorescence phytomers at stages W2.5 and W3.25 but became restricted to the basal region by W4 and W5. Notably, *TSH1* expression persisted in basal ED12 cells at these later stages, indicating continued bract suppression specific to the base of the spike. At W3.25, a small population of cells within a central spikelet expressed *AGL6*, a gene associated with palea identity in wheat (Kong et al. 2022), likely marking the earliest emergence of palea cells (ED15). By W4, central spikelets also contained ED10, ED14, and ED16 domains (associated with stamen and carpel identity), and were enriched for *AG1*, *SEP3-1*, *SEP3-2*, key C- and E-class floral regulators (Cui et al. 2010; Dreni et al. 2011). In contrast, these floral domains were absent from basal spikelets at both stages, reflecting a developmental gradient along the spike and delayed floral progression in the basal region.

### Gene expression patterns differentiate AMs and LRs along the apical-basal axis

Variation in domain composition and gene expression across AMs prior to visible spikelet initiation may reflect shifts in meristem identity along the apical–basal axis of the developing spike. Given limited functional characterization in wheat, we infer putative gene function based on orthologous genes characterized in grasses. At W2.5, central AMs exhibit a transcriptional state distinct from basal AMs, marked by expression of genes including *KNOX5*, *AIL6*, and *HB1.* Orthologs of *KNOX5* (*ZmKNOTTED1*/*OsOSH1*) are expressed in ground tissue and meristems in maize and rice—including in IM, BM, SM, FM—and are excluded from sites of determinate organ initiation (Jackson et al. 1994; Hake et al. 1995; Suzaki et al. 2006). In wheat, *KNOX5* shows a similar pattern yet is notably absent from the meristem corpus of basal AMs. In barley, *HvKN1* is similarly excluded from newly initiated meristems and reactivated upon acquisition of triple SM (TSM) identity, suggesting a role in meristem phase transitions (Demesa-Arevalo et al. 2025). *AIL6*, associated with the indeterminate-to-determinate transition and FM identity (Nole-Wilson et al. 2005; Krizek 2009) and *HB1*, a regulator of FM specification (*LF1* in rice; Zhang et al. 2017), further support transcriptomic signatures of meristem transition in central AMs. Additionally, we observe the specific expression of *TCP24*/*COM1* to AM7,9,10. The barley ortholog *HvCOM1* is expressed in rachilla primordia, and used as a marker of true TSM identity (Demesa-Arevalo et al. 2025). Collectively, these expression patterns indicate that only central AMs acquire SM competency at this stage.

In contrast, the identity of basal AMs remains less clear. Basal AMs are enriched in *RIL1* and *SPL14*, genes associated with BM identity in rice. Knockdown of *OsSPL14* reduces panicle branching and spikelet formation (Wang et al. 2015), while a heterozygous *ril1* mutation in a *ri* background—its close paralog—disrupts the spatial and temporal regulation of BM initiation (Ikeda et al. 2019). BM identity is not directly translatable to the unbranched wheat inflorescence, whereby AMs transition directly to SM identity without branching (Koppolu and Schnurbusch 2019). However, the expression of BM-associated genes in basal AMs may reflect a shared indeterminate state preceding the transition to SM fate. Although the functional consequences of these gene expression patterns have yet to be characterized in wheat, they may help explain phenotypes observed in branching mutants. In spike-type inflorescences such as in wheat and barley, the suppression of branching is regulated by the *COMPOSITUM* pathway (Koppolu et al. 2022). Loss of function in key regulators such as *FRIZZY PANICLE (FZP)* in wheat results in the replacement of spikelets by branch-like structures, most prominent at the base of the spike (Dobrovolskaya et al. 2015; Poursarebani et al. 2015). This branching pattern resembles the panicle-type inflorescence of rice, in which primary branches are longest at the base and gradually decrease toward the apex (Bommert and Whipple 2018). In both species, these patterns suggest that the position and timing of meristem initiation influence the determinacy and fate of lateral organs.

Genetic studies of grasses suggest that suppressed LRs can function as signaling centers regulating adjacent AM activity (Whipple et al. 2010). At stage W2.5, we detect expression of genes in LRs whose orthologs are involved in both bract suppression and promotion of AM development. For example, we observe expression of *TSH1* and *SPL17* in LRs—genes whose orthologs in maize (*tassel sheath1* and *tassel sheath4*, respectively), suppress bract/LR outgrowth while regulating BM determinacy, with *TSH4* acting upstream of *TSH1* (Xiao et al. 2022). Interestingly, *SPL17* expression is restricted to basal LRs and absent from more central and apical LRs (Positions 8 to 13), consistent with the basal specific expression of *RIL1*. Both genes have dual roles, in bract suppression and BM initiation (Ikeda et al. 2019). These results indicate that the genetic program regulating bract suppression and meristem determinacy shifts along the apical–basal axis of the wheat spike. We propose that basal AMs and LRs function as an integrated developmental module, distinct from the central and apical regions, reinforcing the idea of their coordinated role in spike development.

In conclusion, this study establishes a framework for spatial transcriptomic analysis, providing insight into the gene expression programs underlying wheat inflorescence development. As spatially resolved transcriptomics continues to expand in plants, its application across diverse tissues, developmental stages, and species will offer powerful opportunities to uncover the spatio-temporal dynamics of plant development and its diversification across species.

## Materials and methods

### Plant materials

We used 2 BC_6_ near isogenic lines (NIL) differing for *VRT-A2* alleles in a hexaploid wheat (*T. aestivum* cv. Paragon) background. One NIL carried the wildtype Paragon *VRT-A2a* allele, here named *P1^WT^*, whereas the second NIL carried the *VRT-A2b* allele from *T. turgidum* ssp. *polonicum*, *P1^POL^* (Adamski et al. 2021). Plants were grown under a 16/8 h light/dark cycle at 20/15 °C, 65% relative humidity and bottom-watering irrigation (Simmonds et al. 2024).

### Dissections and sample preparation

The *VRT-A2a* NIL was used for semi-spatial RNA-seq, whereas both NILs were used for MERFISH. For semi-spatial RNA-seq we used a published dissection methodology (Faci et al. 2024) to produce basal and central/apical sections. At the EDRe stage (EDR, W2), spikes were bisected, whereas for the LDR (W2.5), LP (W3.25), TS (W4) and CE (W5) stages, the basal section consisted of the most basal 4 spikelets from each spike. Two spikelets were skipped, then the subsequent 4 spikelets were harvested to comprise the central section (Fig. 1A). Samples were stored at −70 °C until RNA was extracted from the pooled microdissected spikes using Qiagen RNeasy Plant Mini and Zymo Direct-zol RNA Microprep kits as described in the manufacturer's manual. Total RNA (∼1 *µ*g) was sent to Novogene UK for PCR-free library preparation and Illumina sequencing (PE150; 50 M reads per sample).

For MERFISH, we used a similar dissection protocol (Faci et al. 2024), but retained the youngest leaves surrounding meristems (Fig. S2). After dissection, meristems were transferred using an RNase-free pipette tip into 4% paraformaldehyde (PFA) in 1× PBS (prepared from 6% formaldehyde [w/v], methanol-free; Pierce 28906) in 2 mL RNase-free Eppendorf tubes. Samples were vacuum infiltrated for 10 min or until tissue sank and incubated overnight at 4 °C. The PFA solution was removed, and the samples were washed 3 times with 1× PBS. Tissue was then immersed in 15% sucrose in 1× PBS at 4 °C for 6 h, followed by immersion in 30% sucrose in 1× PBS at 4 °C overnight.

### Analysis of semi-spatial RNA-seq data

We trimmed raw reads with cutadapt (v1.9.1, Martin 2011) and generated read counts and transcripts per million (TPM) values using Kallisto pseudo-alignment (v0.44.0, Bray et al. 2016) for all genes in the IWGSC RefSeq v1.1 annotation (IWGSC 2014). We conducted subsequent analyses for high confidence gene models with non-zero counts in at least one sample. We transformed read counts (rlog function; DESeq2 v1.34.0; Love et al. 2014), and performed PC analysis with prcomp (R Core Team 2018). We calculated differential expression (*P* < 0.001; Benjamini–Hochberg corrected) between central and basal sections across time using ImpulseDE2 (v3.6.1; Fischer et al. 2018), on genes with average >0.5 TPM for at least one stage-section combination. We clustered the 12,384 differentially expressed genes using *k*-means (k1:10) and displayed with pheatmap (v1.0.12; Kolde 2019). RNA-seq sample metadata, TPM values, and ImpulseDE2 output, see archive folder “RNAseq.tar.gz” at doi.org/10.5281/zenodo.14515926.

### Gene panel selection and design for MERFISH

We designed a 300-gene panel for MERFISH, comprising 200 genes associated with spike development, and 100 genes from a separate wheat grain project which are not described here. We removed genes that could not accommodate at least 25 specific probes, based on Vizgen's probe design software, except for 3 genes targeted by between 20 and 25 probes. MERFISH probes were designed and synthesized by Vizgen.

### Meristem embedding and sectioning

We cleaned all surfaces and dissection tools with RNABlitz before use. We marked a 1 cm × 1 cm area on the back of a Tissue-Tek mold (25 × 20 × 5 mm; Thermo Fisher, AGG4580) and filled with Tissue-Plus OCT compound (Agar Scientific, AGR1180). We also filled a 60 mm Petri dish with OCT. We removed individual meristems from the 30% sucrose solution using clean dissection tools, aided by a drop of OCT on the tool to adhere the meristems during collection. We transferred meristems to the OCT-filled Petri dish, where they were mixed with OCT to remove residual sucrose and ensure complete coating. Using a stereomicroscope (Leica S9 with an HXCAM HiChrome HR4 Lite camera and a Photonic Optics light source), we inspected meristems for air bubbles, which were carefully removed with a fine dissection tool. We trimmed excess vegetative tissue as needed. Meristems were then placed into the OCT-filled Tissue-Tek mold, arranged within the marked 1 cm^2^ region according to genotype and developmental stage. Each OCT block contained 5 to 36 meristems, depending on the developmental stage (Fig. S2), and we imaged them using GX Capture-T software. The OCT blocks were flash-frozen and stored at −70 °C.

We performed sectioning using a Leica CryoStar NX70. All inside surfaces and tools were cleaned with Blitz RNase Spray (Severn Biotech Ltd, 40-1735-05), and a fresh blade (MX35 Ultra Microtome Blade, 3053835) was used for each block. We set the chuck temperature to −20 °C, and the blade temperature to −18 °C. We pre-warmed samples at the back of the cryostat for 30 min before sectioning, and brought the MERSCOPE slides (Vizgen, 20400001) to room temperature. We trimmed OCT blocks to remove excess OCT, mounted to the chuck, and further trimmed until tissue was exposed; 10 *µ*m sections were cut to inspect tissue regions on glass slides. Once we identified the region of interest at the optimal depth and angle, 10 *µ*m sections were flattened with paintbrushes, flipped, and mounted onto room-temperature MERSCOPE slides, following the placement and technique outlined in the MERSCOPE user guide. After mounting, we placed the slides in 60 mm Petri dishes and incubated at the back of the cryostat for 30 min. We then fixed the slides in 4% methanol-free PFA in 1× PBS for 10 min. We washed the slides 3 times with 1× PBS, incubating for 5 min per wash. We removed residual PBS with a pipette and air-dried the slides for 1 h in a cell culture hood with the Petri dish lid closed. We then incubated the slides with 5 mL of 70% ethanol prepared in RNase-free water. Petri dishes were sealed with parafilm and stored at 4 °C, either overnight or for up to 7 d. For more details, see doi.org/10.17504/protocols.io.rm7vzqwb4vx1/v1.

### MERSCOPE workflow

We performed slide preparation following guidelines for non-resistant fixed frozen tissue clearing (91600002_MERSCOPE Fresh and Fixed Frozen Tissue Sample Preparation User Guide_Rev E [Vizgen]). We prepared slides using Vizgen sample preparation kit (Vizgen, 10400012) with all instruments (including hybridization box [Brabantia, 203480]) cleaned using both 70% ethanol and then RNAseZAP (Invitrogen, AM9782) or Blitz RNase Spray (Severn Biotech Ltd, 40-1735-05). We performed checks for autofluorescence at 10 × using an EVOS FL2 microscope under a DAPI light cube, recording light intensity levels to decide on reduction of autofluorescence before and after photobleaching (performed for between 3 and 8 h in EtOH 70%, Vizgen 10100003). A 300-gene probe set (Vizgen product number 20300007) was applied and hybridized for 48 h. On the days of a run, we re-checked autofluorescence levels and placed samples in the photobleacher for an additional 3 h if necessary. After DAPI staining, we also made checks for efficiency of staining. Clearing times varied depending on run slots. A standard clearing at 47 °C for 1 d with clearing buffer (Vizgen) containing proteinase K (NEB, P81070S) was always performed, whereas additional days (1 to 4 d) of clearing at 37 °C without proteinase K in the buffer was performed. Tissue never fully cleared by eye nor when using a light microscope before MERSCOPE runs.

Upon imaging, care was taken to minimize smears and lint on the slide by cleaning with 80% ethanol and lens cleaning tissue (2105-841, Whatman). We outlined regions of interest around individual spikes on the slide overview using DAPI staining (Fig. S2). Following 60 × imaging, we decoded transcripts using the panel specific MERSCOPE Codebook. We processed raw data with the MERSCOPE Instrument Software to generate and output file structures as described in MERSCOPE Instrument User Guide.

### Cell segmentation and processing

We performed cell segmentation on stitched images of DAPI and PolyT staining. Prior to segmentation and to minimize error, we lightened seam lines in the stitched images using FIJI (v 1.54f; Schindelin et al. 2012). Dark stitching lines were processed using the following steps: “Process > Filters > Maximum” (radius = 2 pixels, applied twice) and “Process > Filters > Median” (radius = 2 pixels, applied twice). Then applied 3 times across the entire image: “Process > Filters > Gaussian Blur” (radius = 4 pixels). See Fig. S3 for image edits and segmentation results.

We performed cell segmentation and transcript assignment using the Vizgen Post-Processing Tools (v1.2.2; Wiggin and Yu 2024), within a Python virtual environment on Ubuntu 20.04. We used Cellpose2 cyto2 model (Pachitariu and Stringer 2022) with DAPI (blue channel) as the nuclear marker and PolyT (green channel) as the cytoplasmic marker. For segmentation parameters, see logs (doi.org/10.5281/zenodo.14515926). We exported segmentation results as polygon geometry in both mosaic and micron space, assigned transcripts to cell boundaries using the partition-transcripts function in VPT, and generated cell metadata with the derive-entity-metadata function. Cell metadata includes a unique Entity ID, center *X*,*Y* coordinates, and volume of the cell (*µ*m^3^; 2D segmented area × tissue section thickness). Finally, we integrated cell boundaries into existing .vzg files for visualization in the Vizgen MERSCOPE Visualizer Tool (MERSCOPE Vizualizer 2023) using the update-vzg function. All implementation scripts are available (https://github.com/katielong3768/Wheat-Inflorescence-Spatial-Transcriptomics) with example commands. For a gene × cell matrix for all samples, see archive folder “QC.tar.gz” at doi.org/10.5281/zenodo.14515926.

### Quality checks and filtering

We loaded the spatial transcriptomic data from the 8 samples into AnnData objects (anndata v0.10.7; Virshup et al. 2021) and processed using Squidpy (v1.4.1; Palla et al. 2022) and Scanpy (v1.10.0; Wolf et al. 2018). We filtered expression data to include the 200 spike development and control genes, selected cells from a single inflorescence within the imaged area and excluded low-quality cell segmentations transcript count (<25 counts). To reduce segmentation noise, a volume threshold was applied to remove small non-cellular artifacts produced during Cellpose2 segmentation (<500 *µ*m^3^).

For each sample we calculated QC metrics (total counts per cell, number of genes detected per cell, percentage of counts from top-expressed genes). We assessed error rate using total counts per cell in 15 “blank” barcodes (described in Chen et al. 2015), and off-target binding across wheat homoeologs. Finally, we normalized expression data for all samples using scanpy functions sc.pp.normalize_total (normalizes each cell by total counts over all genes, so all cells have same total count after normalization) and sc.pp.log1p (logarithmizes the data matrix) (v1.10.0, Wolf et al. 2018). Supplementary information including QC metrics per sample and total blank counts per sample, can be accessed in archive folder “QC.tar.gz” at doi.org/10.5281/zenodo.14515926.

As a further QC metric, we performed in silico spike dissections equivalent to those captured by physical microdissection for 8 high-quality MERFISH sections using the MERSCOPE Visualizer “draw ROI polygon” tool (MERSCOPE Vizualizer 2023). We extracted cell IDs within each selected region and summated the total normalized counts, yielding transcript count information on 200 genes total. We used Spearman's rank correlation coefficients between these values and the mean TPM values from the relevant genotype-section-stage combination of the semi-spatial RNA-seq data (Fig. S7). For further information on ROI areas and correlation results, see archive folder “QC.tar.gz” at doi.org/10.5281/zenodo.14515926.

Additionally, we identified in situ hybridization results in wheat, barley (*H. vulgare*), rice (*O. sativa*), and maize (*Z. mays*) from equivalent tissues and time points as those used for MERFISH and visualized them in side-by-side comparisons as described in “Staining, Segmentation, and Transcript Visualisation” (Fig. S8).

### Staining, segmentation, and transcript visualization

We processed the cell segmentation data as GeoDataFrames (Geopandas v0.14.4; Jordahl et al. 2020), and converted the transcript coordinates into a GeoDataFrame from global *x*- and *y*-coordinates. We performed a spatial join operation to assign transcripts to segmented cells, retaining only transcripts located within cell boundaries. We loaded the DAPI staining image as a .tiff file, alongside a transformation matrix enabling conversion between pixel space to physical (micron) space, as generated by the MERSCOPE Instrument Software. The transformation matrix converted to micron space and applied the raw image using Scikit-image (v0.23.3, van der Walt et al. 2014). After transformation, the image was normalized, and image contrast and brightness were adjusted with Scikit-image. We next rotated segmented cell polygons and transcript coordinates using NumPy (v1.26.3, Harris et al. 2020), and visualized cell geometries as polygons using Matplotlib (v3.8.2, Hunter 2007) with polygon handling and transformations facilitated by Shapely (v2.0.4, Gillies et al. 2022). Transcripts were overlaid as point features. Note that visualized transcripts represent a single detection of a transcript from the MERFISH run, and do not represent normalized transcript counts. The corresponding image was rotated with Scipy.ngimage (Virtanen et al. 2020; v1.13.0). Full details in implementation scripts, see https://github.com/katielong3768/Wheat-Inflorescence-Spatial-Transcriptomics.

### MERFISH data integration, unsupervised clustering, and gene enrichment analysis

We processed spatial transcriptomic data from 8 samples (4 timepoints; 2 NILs) using the Scanorama (Hie et al. 2019, 2024) integration tool, and performed clustering using the Leiden algorithm with a resolution parameter of 1.0. Spatial maps of Leiden cluster assignment were performed as described in “Staining, Segmentation, and Transcript Visualisation.” We exported the ED assignment of each per cell (see archive folder “SampleIntegration_Clustering.tar.gz” at doi.org/10.5281/zenodo.14515926). The number of domains representing each sample were summated, with domains representing <0.5% of total cells across a sample removed. Next, we performed gene enrichment analysis on the integrated AnnData object with Scanpy function sc.tl.rank_genes_groups() using the logistic regression model (Ntranos et al. 2019). This analysis returned a ranked list of genes most probable to be enriched gene markers, which we displayed alongside the average normalized expressions per ED for each sample. We determined top enriched values (using a + 2 SD threshold) and used these to annotate EDs with tissue type identity labels. See archive folder “GeneEnrichmentAnalysis.tar.gz” at doi.org/10.5281/zenodo.14515926 for domain assignments, gene enrichment analysis output, and domain annotations.

### Transect analysis of *VRT-SEP* gradients

We filtered cells from 2 samples (W4, *VRT-A2a* and *VRT-A2b NILs*) to include only cells from the inflorescence region. We defined the inflorescence boundary by the first suppressed LR (ED12). These cells were selected in the MERSCOPE Visualizer tool (MERSCOPE Vizualizer 2023) using the Polygon Lasso Tool, exported as a .csv file, and the segmented cells and transcripts were mapped as previously described. The *y*-axis of the spatial plot was divided into 30 transverse bins along the spike. Each cell was assigned to a bin based on the *y*-coordinate of its center, and we averaged the normalized transcript counts per cell within each bin (Fig. 4a and B). For both samples, we binarized gene expression data for *VRT2* and *SEP1-4* within each cell, assigning a value of 1 for detected reads and 0 for no detected reads. For each ED, we quantified the number of cells expressing only *VRT2*, only *SEP1-4*, or co-expressing both genes and visualized them as a percentage with Matplotlib (Hunter 2007).

### Gene expression analysis on AMs and LRs

We selected LDR (W2.5) and LP (W3.25) *P1^WT^* inflorescence cells using the MERSCOPE Visualizer Polygon Lasso tool and exported cell identity data as a .csv file. We defined the inflorescence boundary by the first suppressed LR (ED12) and excluded cells outside the inflorescence. Cell counts were summed by ED, and we calculated the cumulative percentage of cells in the most populated ED to assess their contribution to the total cell population. We calculated the top EDs accounting for ∼94% of the cells in the sample.

Groups of cells comprising the LRs and AMs (defined by EDs) were annotated as “Custom Cell Groups” in the MERSCOPE Visualizer tool (MERSCOPE Vizualizer 2023). We delineated AM boundaries by ED4 cells along the adaxial and abaxial axes, extending to the start of ED3 cells along the medio-lateral axis. We identified LRs as groups of ED12 cells beginning beneath the end of ED4 cells from adjacent AMs. We labeled AMs and LRs sequentially from 1 (most basal) to 13 (most apical) along the inflorescence (Fig. 5c and H). We calculated the total number of cells in basal (LR1-4) and central (LR8-11) LRs in sample W2.5 *P1^WT^* and determined the mean cell numbers and summary statistics to compare ridge sizes between these regions. This process was repeated for spikelets in samples W3.25 *P1^WT^*, W3.25 *P1^POL^*, W4 *P1^WT^*, W4 *P1^POL^*, following the same criteria of cells alternating between LRs (ED12), and extending across the lengths of ED4 cells along the adaxial-abaxial axis (Fig. S12).

Normalized gene expression values per cell were averaged by LR or AM group and filtered to include only genes with at least one average expression score above 0.30 across all groups. We standardized the resulting data matrix using StandardScaler and performed PC analysis with scikit-learn (v1.4.2; Pedregosa et al. 2011), to extract the first 2 PCs (Fig. 5D and I). We inverted PC1 and PC2 scores to align the axes with the desired biological orientation and calculated the Spearman's correlation coefficient between the position along the inflorescence (1 to 13) and PC1 scoring (Scipy.stats, v1.13.0). We extracted the top genes contributing to PC1 through PC1 loading scores, and calculated average expression (*Z*-score normalized) in each cell grouping and visualized using Matplotlib (v3.8.2; Hunter 2007) and Seaborn (v0.13.1; Waskom 2021). All scripts and supplementary data are available (https://github.com/katielong3768/Wheat-Inflorescence-Spatial-Transcriptomics/ and doi.org/10.5281/zenodo.14515926).

### Accession numbers

Wheat (*AG1*, TraesCS1D02G127700; *AG2*, TraesCS3A02G314300; *AGL14*, TraesCS3D02G284200; *AGL6*, TraesCS6A02G259000; *AIL6*, TraesCS4A02G123800; *AP2-5*, TraesCS5A02G473800; *AP3*, TraesCS7A02G383800; *FDH*, TraesCS4D02G296400; *FZP*, TraesCS2B02G136100; *HB1*, TraesCS5A02G549700; *ILI3*, TraesCS4A02G016000; *KNOX5*, TraesCS4A02G256700; *LEC1*, TraesCS6A02G287300; *MND1*, TraesCS7B02G413900; *MT2B*, TraesCS1B02G042200; *PI1*, TraesCS1A02G264300; *PI2*, TraesCS3A02G406500; *Roc3t*, TraesCS1D02G197300; *Roc7t*, TraesCS7A02G308400; *SEP1-1*, TraesCS4B02G245800; *SEP1-2*, TraesCS4D02G243700; *SEP1-4*, TraesCS7D02G120500; *SEP1-5*, TraesCS7D02G120600; *SEP1-6*, TraesCS5B02G396700; *SEP3-1*, TraesCS7D02G261600; *SEP3-2*, TraesCS5A02G286800; *SP3*, TraesCS5A02G401800; *SPL14*, TraesCS7A02G246500; *SPL17*, TraesCS5A02G265900; *TSH1*, TraesCS1A02G418200; *VRT2*, TraesCS7A02G175200; *YABBY1*, TraesCS1D02G162600; *YABBY7*, TraesCS6D02G220400), barley (*HvCOM1*/*HvTCP24*, HORVU.MOREX.r3.5HG0479720; *HvKN1*, HORVU.MOREX.r3.4HG0339120), rice (*ONI1*, Os03g0181500; *OSH1*, Os03g0727000; *Roc3t*, Os10g0575600; *Roc7t*, Os08g0136100; *TOB2*, Os02g0643200), and maize (*knotted1*, Zm00001eb055920; *tassel sheath1*, Zm00001eb296770; *tassel sheath4*, Zm00001eb316740). Table S1 includes the accession numbers in wheat, barley, rice, and maize for all genes used in the design.

## Supplementary Material

koaf282_Supplementary_Data

## Data Availability

Code associated with this project is available at Github including all implementation scripts (https://github.com/katielong3768/Wheat-Inflorescence-Spatial-Transcriptomics). All files, images, and segmentation outputs used in the analyses and described in GitHub are available on an individual sample basis (https://zenodo.org/records/15720855). The RNA-sequencing data generated in this study were submitted to NCBI under BioProject number PRJNA1201104. Visualization of transcript counts per cell for *P1*^WT^ samples are available at www.wheat-spatial.com.

## Dive Curated Terms

The following phenotypic, genotypic, and functional terms are of significance to the work described in this paper:

LEC1 Gramene: AT1G21970
LEC1 Araport: AT1G21970

SPL14 Gramene: AT1G20980
SPL14 Araport: AT1G20980

FDH Gramene: AT5G14780
FDH Araport: AT5G14780

DAPI CHEBI: CHEBI:51231

AIL6 Gramene: AT5G10510
AIL6 Araport: AT5G10510

AP3 Gramene: AT3G54340
AP3 Araport: AT3G54340

MT2b Gramene: Os05g0111300
MT2b Araport: Os05g0111300

OSH1 Gramene: Os03g0727000
OSH1 Araport: Os03g0727000

AGL6 Gramene: HORVU.MOREX.r3.6HG0604360
AGL6 Araport: HORVU.MOREX.r3.6HG0604360

CUC3 Gramene: AT1G76420
CUC3 Araport: AT1G76420

spikelet AmiGo: PO:0009051 AmiGO2
