# Supplementary material for: Spatial transcriptomics reveals expression gradients in developing wheat inflorescences at cellular resolution
Source: Plant Cell. 2025 Dec 13;38(1):koaf282. doi: 10.1093/plcell/koaf282 (PMC12776951; doi:10.1093/plcell/koaf282)
Supplement: koaf282_Supplementary_Data [file koaf282_supplementary_data.zip › Supplementary Tables Legends.docx]

**Supplementary Table S1.** Composition of 200-gene MERFISH panel and expression across developing wheat inflorescences.

**Supplementary Table S2.** Enriched genes in the 18 expression domains.
