# Supplementary material for: Spatial transcriptomics reveals expression gradients in developing wheat inflorescences at cellular resolution
Source: Plant Cell. 2025 Dec 13;38(1):koaf282. doi: 10.1093/plcell/koaf282 (PMC12776951; doi:10.1093/plcell/koaf282)
Supplement: koaf282_Supplementary_Data [file koaf282_supplementary_data.zip › Long et al_Supplementary Data_Dec2025_KL_tracked.pdf]

Supplementary Data for *Spatial Transcriptomics Reveals Expression Gradients in Developing Wheat Inflorescences at Cellular Resolution*

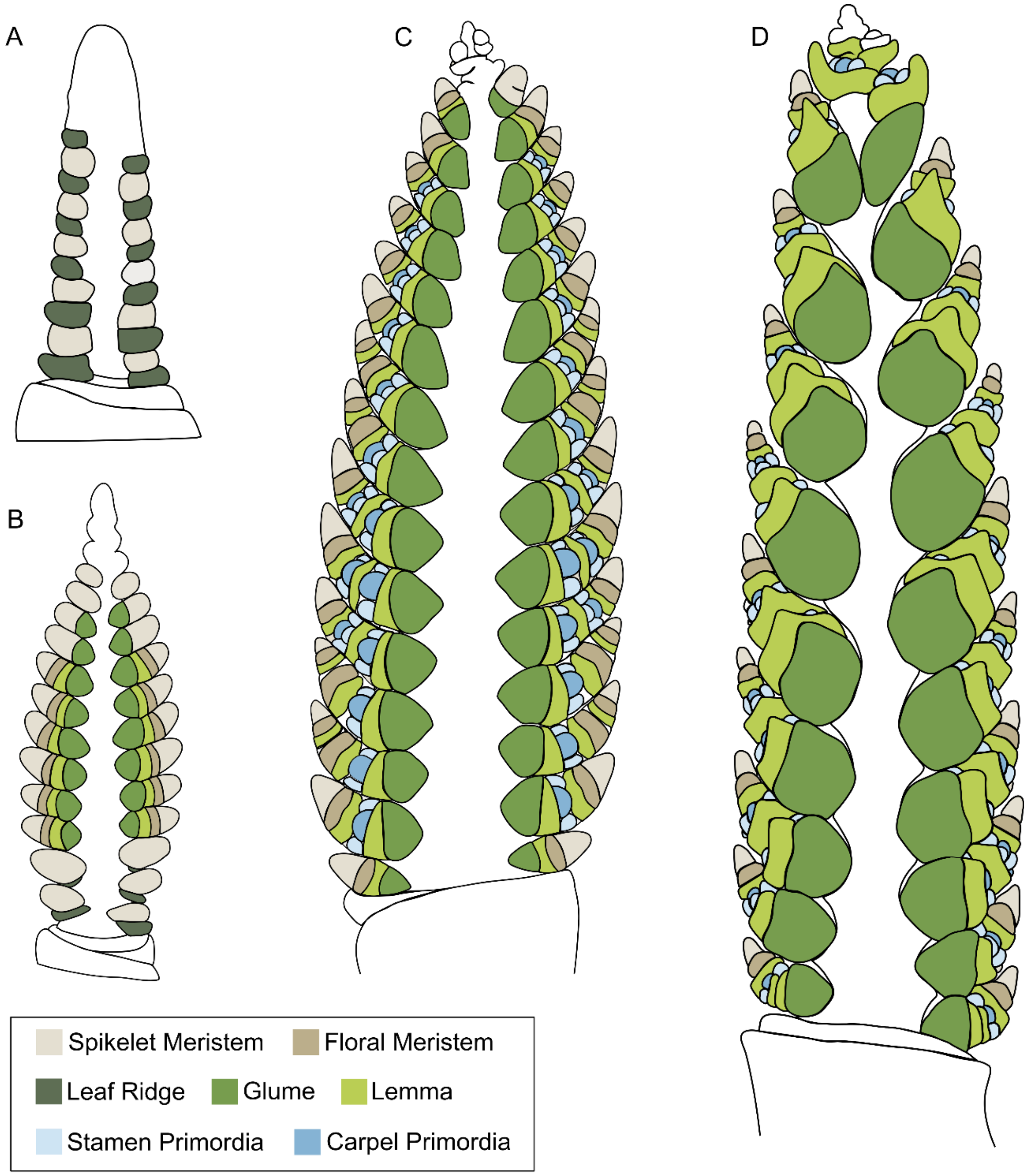

Supplementary Figure S1. Anatomical diagram of wheat inflorescence stages sampled for MERFISH, representing **A)** W2.5 (Late Double Ridge). Inflorescence meristem initiates set of paired ridges, the axillary meristem (termed spikelet ridge) and lower leaf ridge, formed in a distichous phyllotaxis. **B)** W3.25 (Lemma Primordia). Central spikelets have formed glume primordia ridge and lemma primordia ridge. The spikelet meristem is initiating floral meristems. **C)** W4 (Terminal Spikelet). In the central spikelets, stamen and carpel primordia are differentiating in lower florets. **D)** W5 (Carpel Extending Round). Carpel primordia have formed and in floret 1, carpels are extending around three sides of ovule. Glumes enfold lower florets. All staging information sourced from Waddington and Cartwright 1983; Kirby and Appleyard 1984. Diagrams were adapted from the tracings of light images of dissected wheat inflorescences, representative of samples used in MERFISH workflow (images taken on Leica S9 stereomicroscope). Stages not to scale. Supports Figures 1-5.

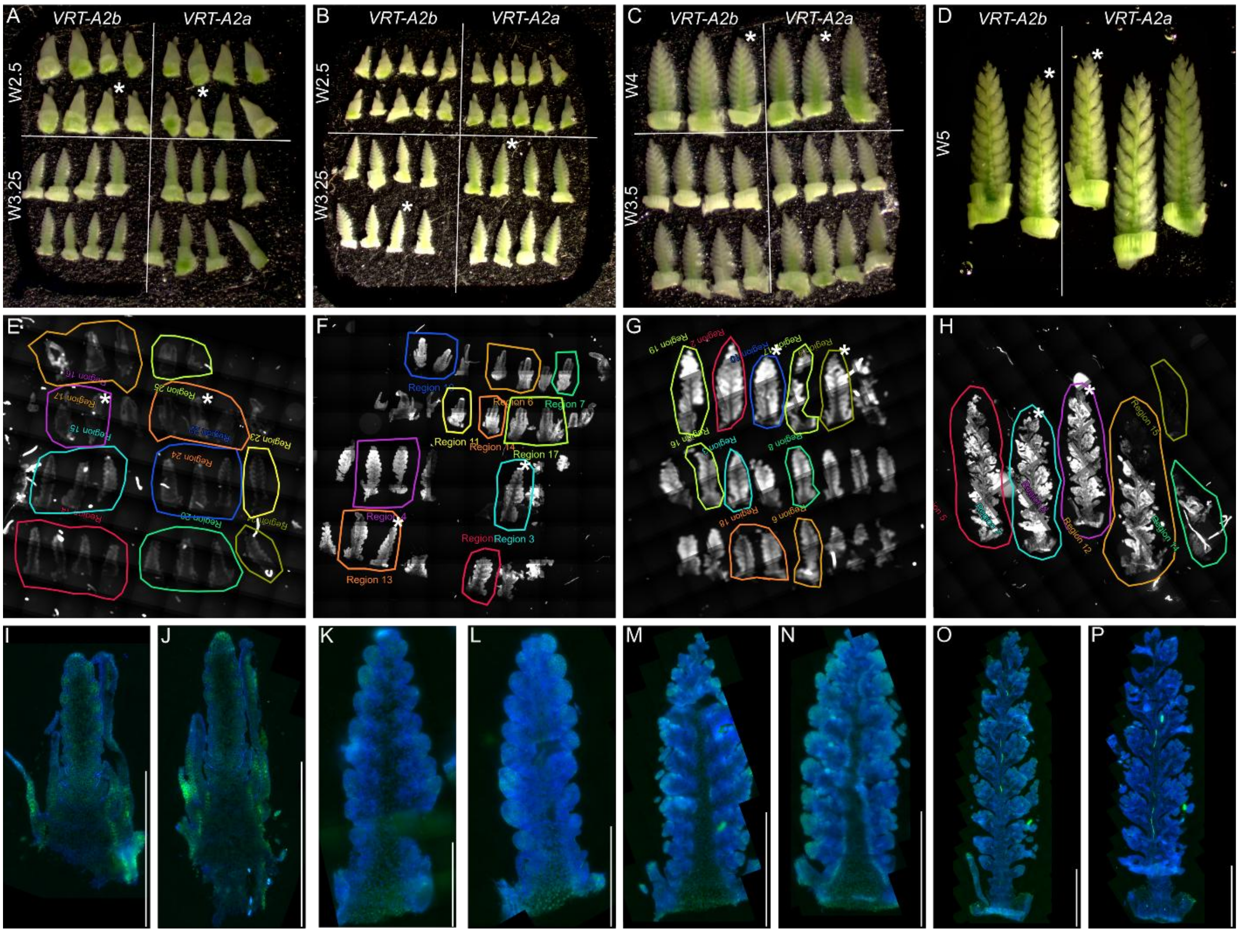

Supplementary Figure S2. Optimal cutting temperature (O.C.T.) block layout, ‘Region of Interest’ selections, and eight cryosections across four MERSCOPE experimental runs. Black lines bordering samples indicate a 1 cm x 1 cm area, marked in black marker pen on the back of the plastic mold in which the samples are sitting in (see Methods). **A-D)** Layout of wheat inflorescences in OCT blocks with genotype and developmental stage annotations. Asterisks denote spikes selected for final analyses. Taken on Leica S9 stereomicroscope. **E-H)** DAPI stain overview and experimental region selections from MERSCOPE Instrument output. **I-P)** Eight selected cryosections for onward analysis, stages W2.5 **(I-J)**, W3.25 **(K-L)**, W4 **(M-N)**, W5 **(O-P)** in genotypes $P1^{WT}$ **(I,K,M,O)** and $P1^{POL}$ **(J,L,N,P).** Stained images taken on MERSCOPE instrument prior to MERFISH experimental run, and visualised on the MERSCOPE Visualizer Tool (See Methods). DAPI = Blue, PolyT = Green**.** The images shown in panels **(E-P)** are composite images. Additional images of cryosections in panels **(I-P)** are shown in Supplementary Figures S3, S4, S8, S10, S11, S12. Supports Figure 1.

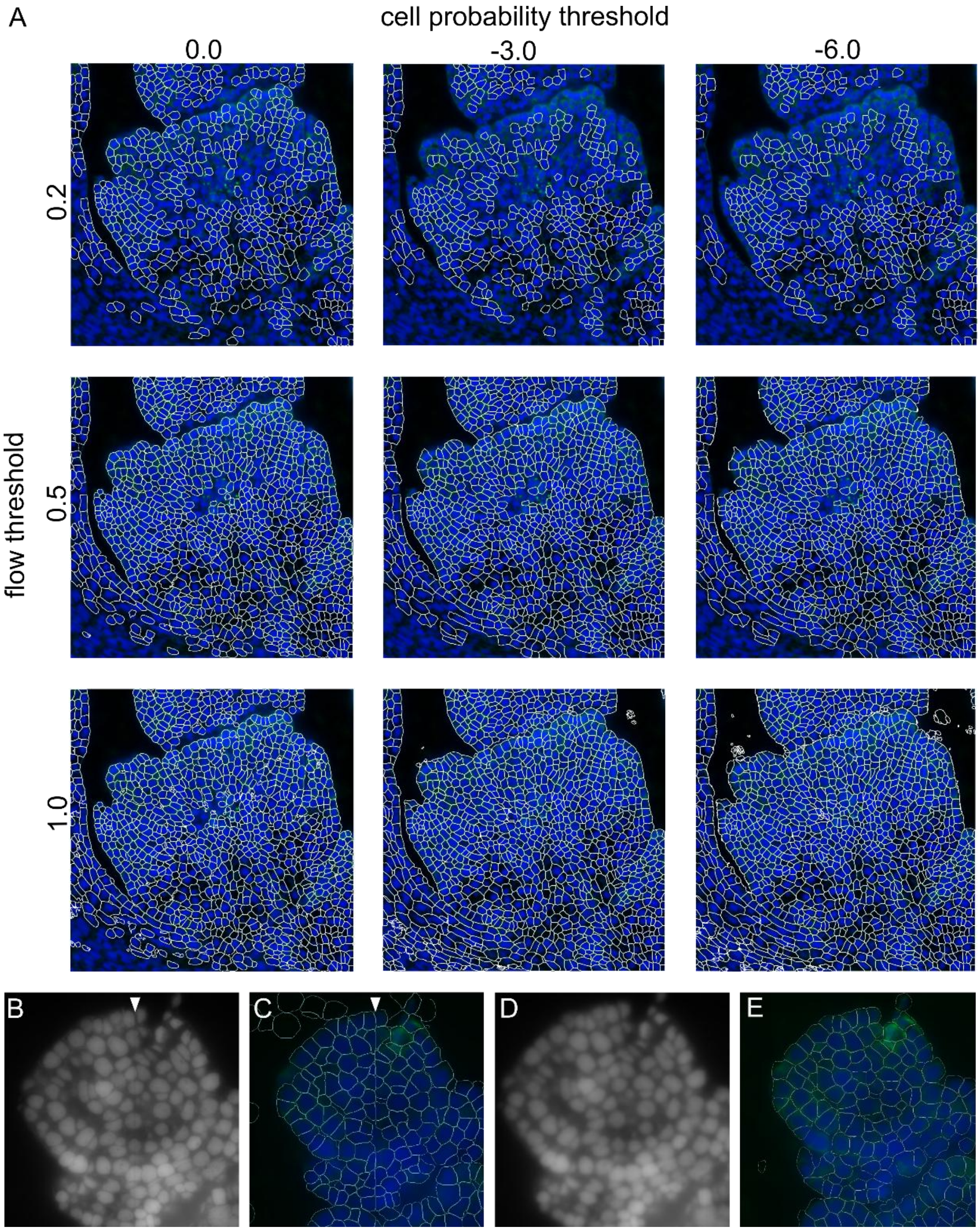

Supplementary Figure S3. Optimization of cell segmentation parameters and editing of seam lines in stained images improves cellular segmentation outputs. **A)** Segmentation outputs with parameters of flow threshold (0.2-1.0) and cell probability threshold (-6.0-0.0) in cellpose2 on wheat spikelet tissue (Pachitariu and Stringer 2022), identifying an optimal flow threshold of 1.0 and a cell probability threshold of –3.0. **B)** Raw DAPI stain, as output from MERSCOPE Instrument. **C)** Cellpose2 segmentation outputs prior to image edits, visualised in Vizgen MERSCOPE Visualiser Tool. White arrow denotes seam line detected through segmentation resulting in false cell boundaries. **D)** Image J filters (Maximum Filter, x3 and Median Filter, x3, Gaussian blur, See Methods) applied to DAPI stain image. **E)** Cell segmentation of edited DAPI stain image, visualised in Vizgen MERSCOPE Visualiser Tool. DAPI = blue, PolyT = green, cell segmentation boundaries in white. Supports Figure 1.

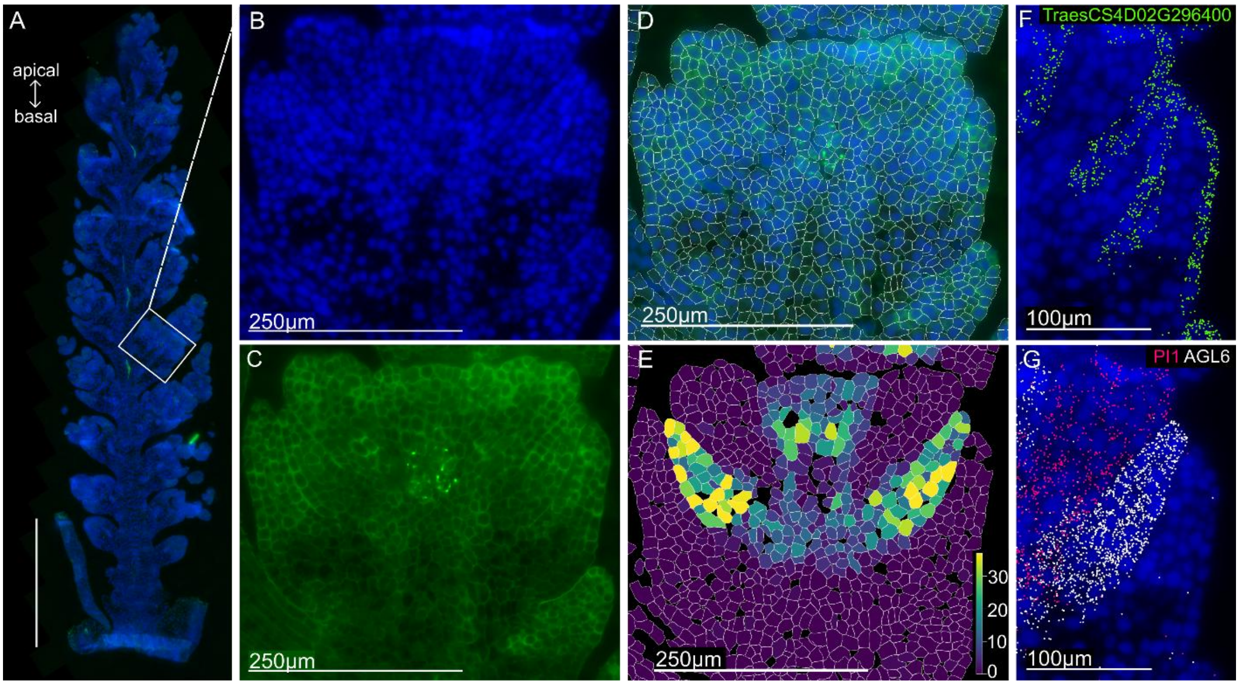

Supplementary Figure S4. Cell segmentation and transcript assignment of 200-gene MERFISH panel in wheat inflorescence tissue**. A)** Cryosection of W5 spike showing DAPI stain (blue) and polyT stain (green). Inset: higher magnification of floral tissues with individual staining of DAPI (**B**) and PolyT (**C**). **D)** Cellular segmentation with cellpose2, filtered for high quality cells. **E)** Heatmap displaying 'transcript counts per cell for *AGL6*, with assignment conducted using the Vizgen Post-Processing Tool. **F)** *TRAESCS4D02G296400* transcripts (green) localized in the first cell layer of floral tissues. **G)** Tissue-specific expression patterns of *AGL6* (white) and *PI1* (pink) with distinct, non-overlapping spatial localization towards tips of palea and stamen, respectively. Supports Figure 1.

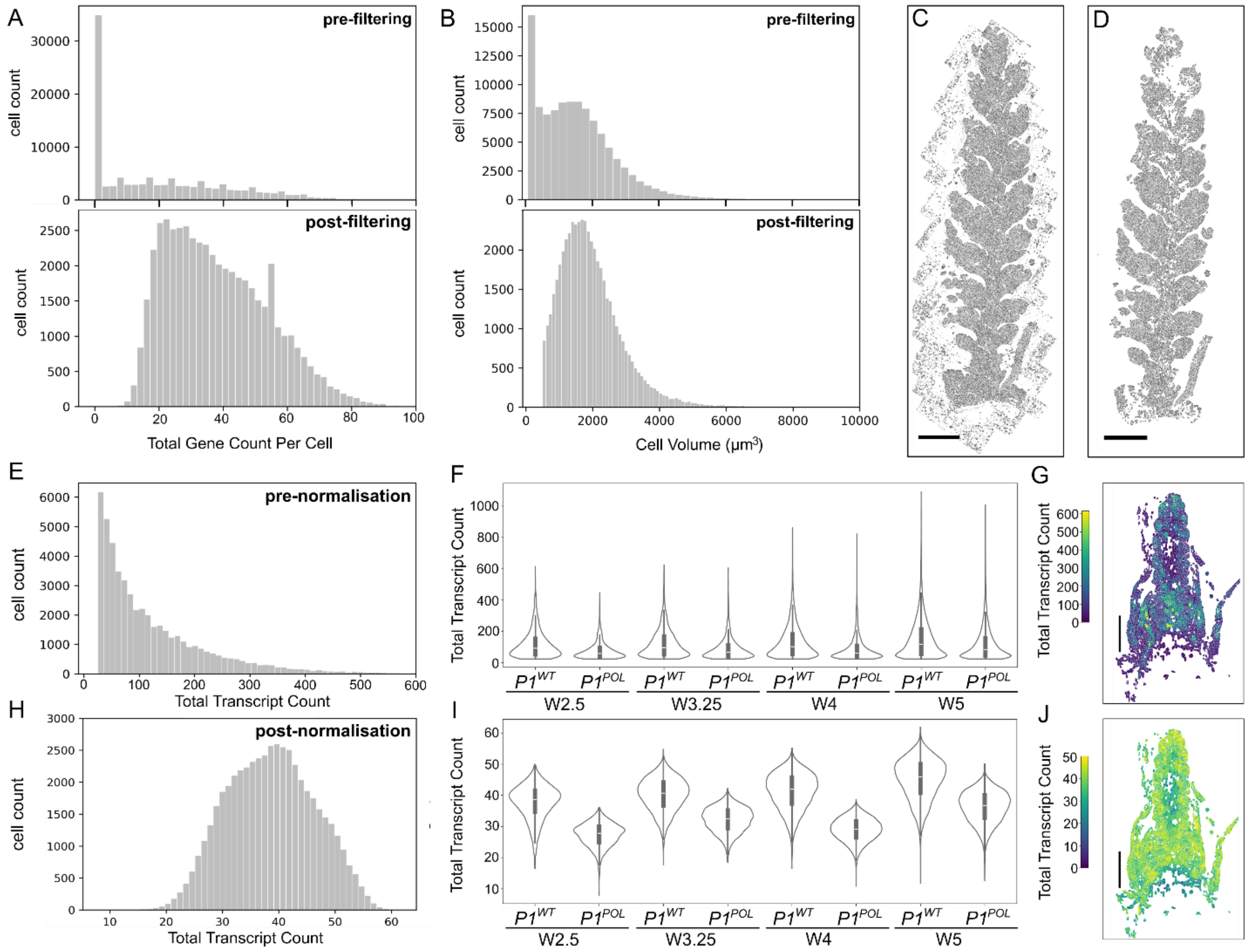

Supplementary Figure S5. Filtering of low-quality cells and normalization of transcript count in eight selected regions of interest. **A)** Total gene count per cell before and after filtering low quality cells (< 25 transcript counts per cell), combined across eight samples. **B)** Cell volume before and after filtering small segmentation artifacts (< 500 μm^3) **C-D)** Cellular segmentation in cellpose2 before (**C**) and after (**D**) filtering of small segmentation artifacts in sample W5, $P1^{WT}$. **E-G)** Total transcript count per cell, before counts normalization; displayed in (**E**) frequency plot of eight samples combined, (**F**) violin plots of individual samples, (**G**) heat plot of total transcripts per cell displayed over cellular segmentation in sample W2.5, $P1^{WT}$. **H-J)** Total transcript count per cell, after counts normalization with Scanpy functions pp.normalize_total() and pp.log1p(); displayed in (**H**) frequency plot of eight samples combined, (**I**) violin plot of individual samples, (**J**) heat plot of total transcripts per cell displayed over cellular segmentation in sample W2.5, $P1^{WT}$, displaying the reduction of within-sample variability of total transcript count per cell. Supports Figures 1-2.

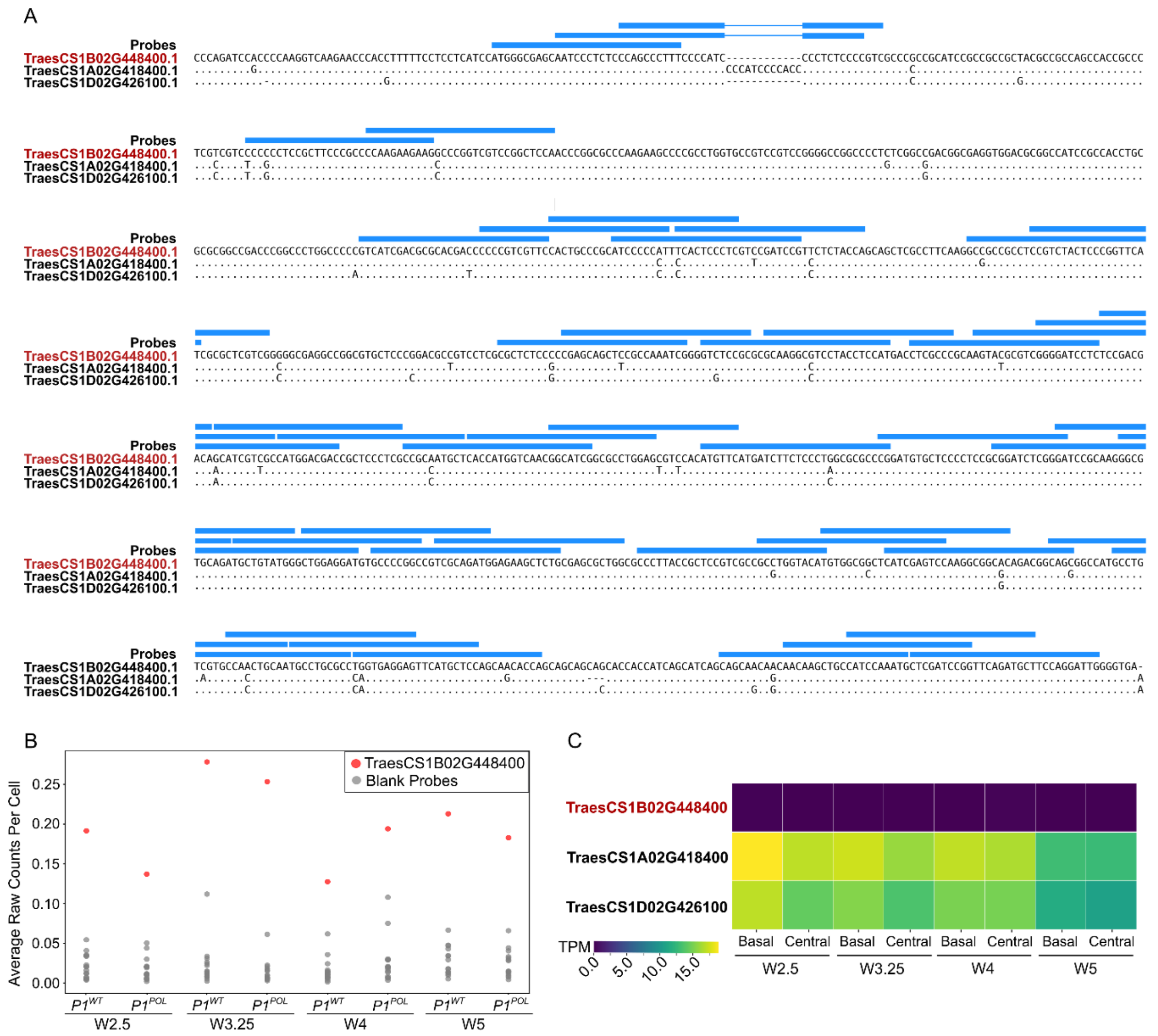

Supplementary Figure S6. MERFISH probes display non-homoeologous binding activity. **A**) Sequence alignment of homoeologous triad *TraesCS1B02G448400*, *TraesCS1A02G418400*, and *TraesCS1D02G426100* using MUSCLE (v5, Edgar 2022). Probes were designed to *TraesCS1B02G448400*; blue bars indicate individual probe target sites along the transcript. Not that probes purposedly targeted polymorphic regions among homoeologs. **B**) Average counts per cell (pre-normalization) of *TraesCS1B02G448400* compared to 15 blank probes across eight samples. **C)** Transcripts per million (TPM) of the homoeologous triad from RNA-seq of microdissected basal and central spike regions at stages W2.5–W5. Supports Figure 1.

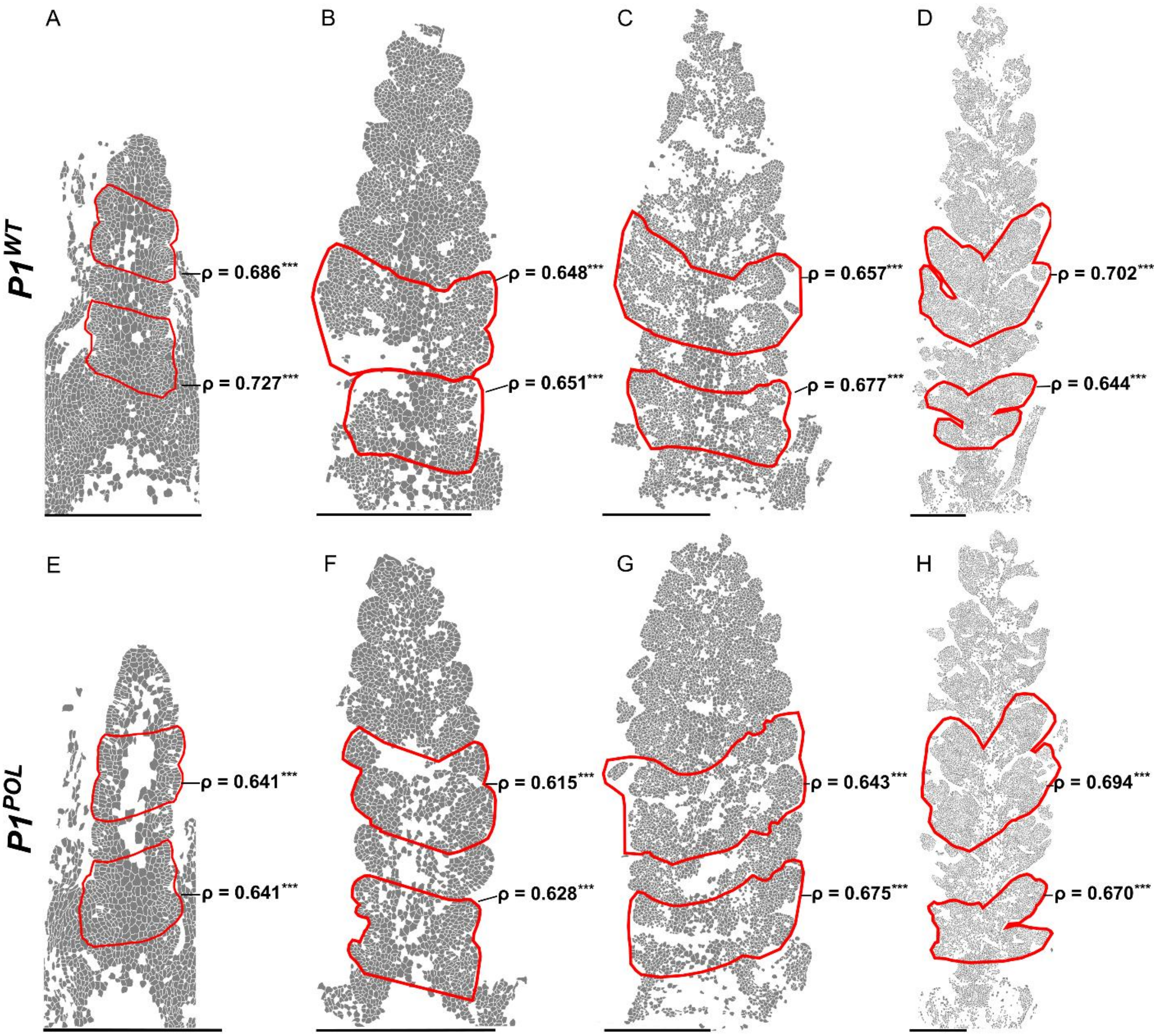

Supplementary Figure S7. Normalized counts from *in silico* dissections of MERFISH regions show strong correlation with TPM expression values from corresponding RNA-seq microdissection regions. (**A-H)** Cell segmentations (grey) overlaid with *in silico* dissection regions (red), as selected using the MERSCOPE Visualizer tool. Spearman's rank correlation coefficients (ρ) are shown for each genotype, developmental stage, and section; significance is indicated (*** $P \leq 0.001$). Supports Figure 1.

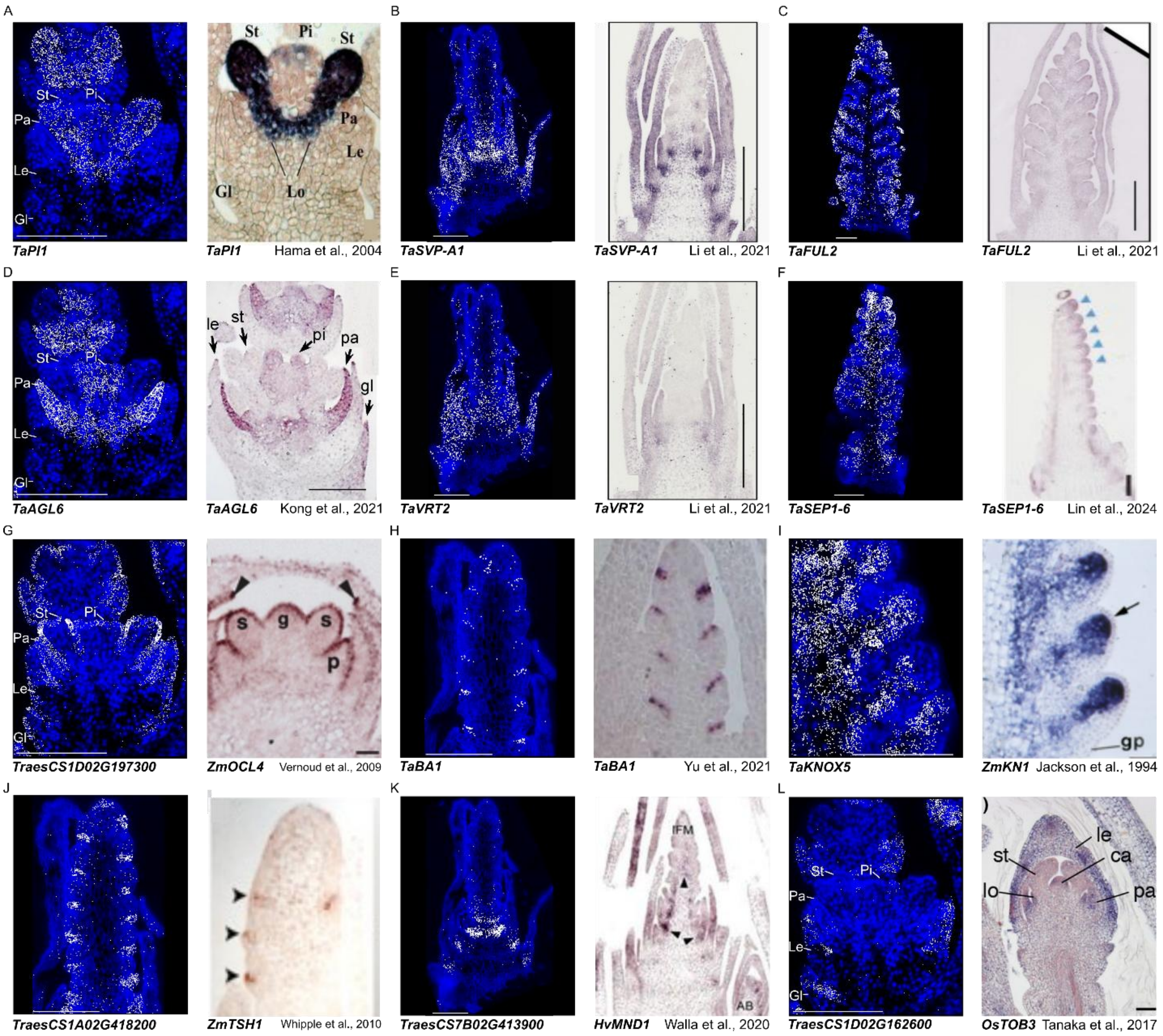

Supplementary Figure S8. MERFISH recapitulates previous *in situ* hybridization results from equivalent inflorescence tissues across multiple cereals. **A-L)** Transcript localisation of wheat genes in MERFISH *P1*^{WT} samples (left) compared to *in situ* hybridization of wheat gene (*Ta)* or cereal ortholog *(Os* = rice*; Hv* = barley*; Zm* = maize) at equivalent inflorescence stage in published studies (right). The image in panel 8A is the same as Figure 3C. The image in panel 8D is the same as Figure 2K and Figure 3H. The image in panel 8J is the same as panel 3M. The image in panel 8K is the same as Figure 3P Supports Figure 1.

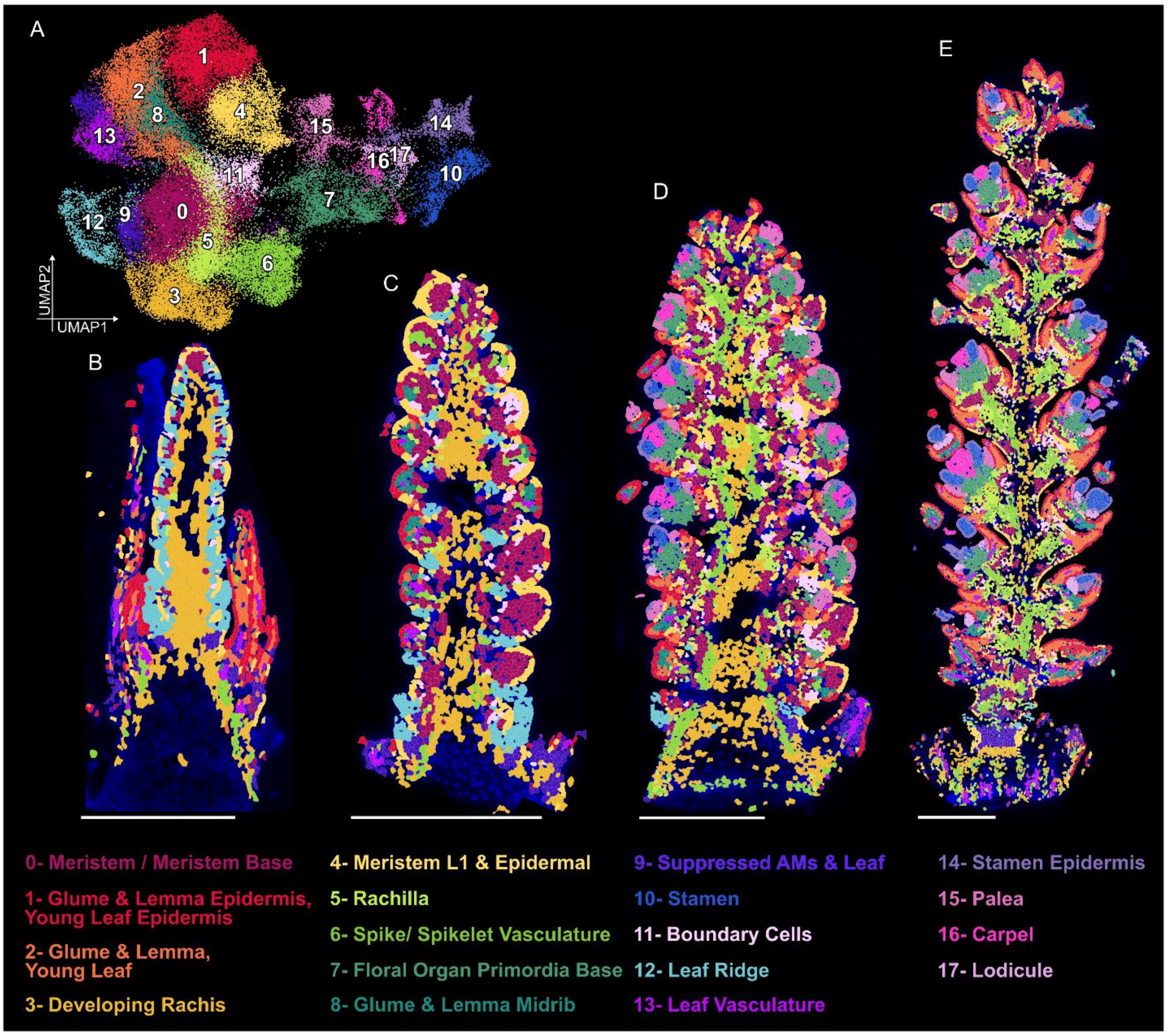

Supplementary Figure S9. Additional replicates in $P1^{POL}$ display consistent patterns of 18 expression domains mapped over four developmental stages. **A)** UMAP projection of cells from eight samples, and expression domain assignment. **B-E)** Spatial maps in $P1^{POL}$ of Leiden clustering across time points W2.5 **(B)**, W3.25 **(C)**, W4 **(D)**, and W5 **(E)** using Squidpy (v1.4.1), Scanpy (v 1.10.0), and Scanorama (v1.7.4) scale bar = 500μm. Supports Figure 2.

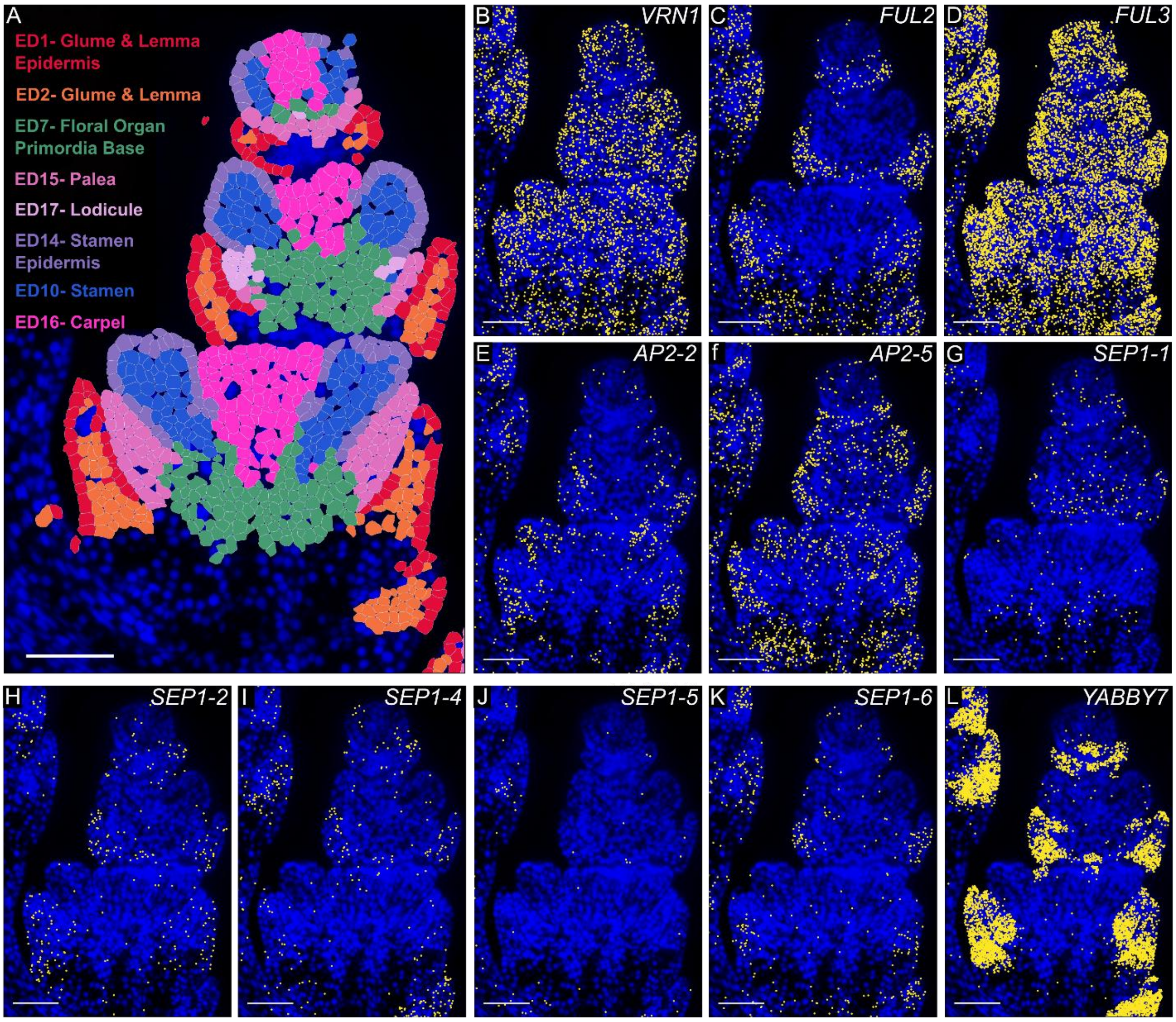

Supplementary Figure S10. Gene expression analysis in floret tissues of additional genes in the ABCDE model of floral development. **A)** Eight expression domains in W5 florets (*P1*^{WT}) mark floral organ identity (ED1,2 = glume/lemma, ED7 = floral organ primordia base, ED15 = palea, ED17 = lodicule, ED14,10 = stamen, ED16 = carpel). **B-L)** Spatial transcript localisation in W5 florets, **(B)** *VERNALIZATION1* (*VRN1*), **(C)** *FRUITFULL 2* (*FUL2*), **(D)** *FRUITFULL 3* (*FUL3*), **(E)** *AP2-LIKE 2* (*AP2-2*), **(F)** *AP2-LIKE 5* (*AP2-5*). **(G-K)** *SEPALLATA 1 (SEP1)* orthologs; **(G)** *SEP1-1,* **(H)** *SEP1-2,* **(I)** *SEP1-4,* **(J)** *SEP1-5,* **(K)** *SEP1-6.* **(L)** *YABBY7.* Scale bar = 100 µm. Blue stain = DAPI. Supports Figure 3.

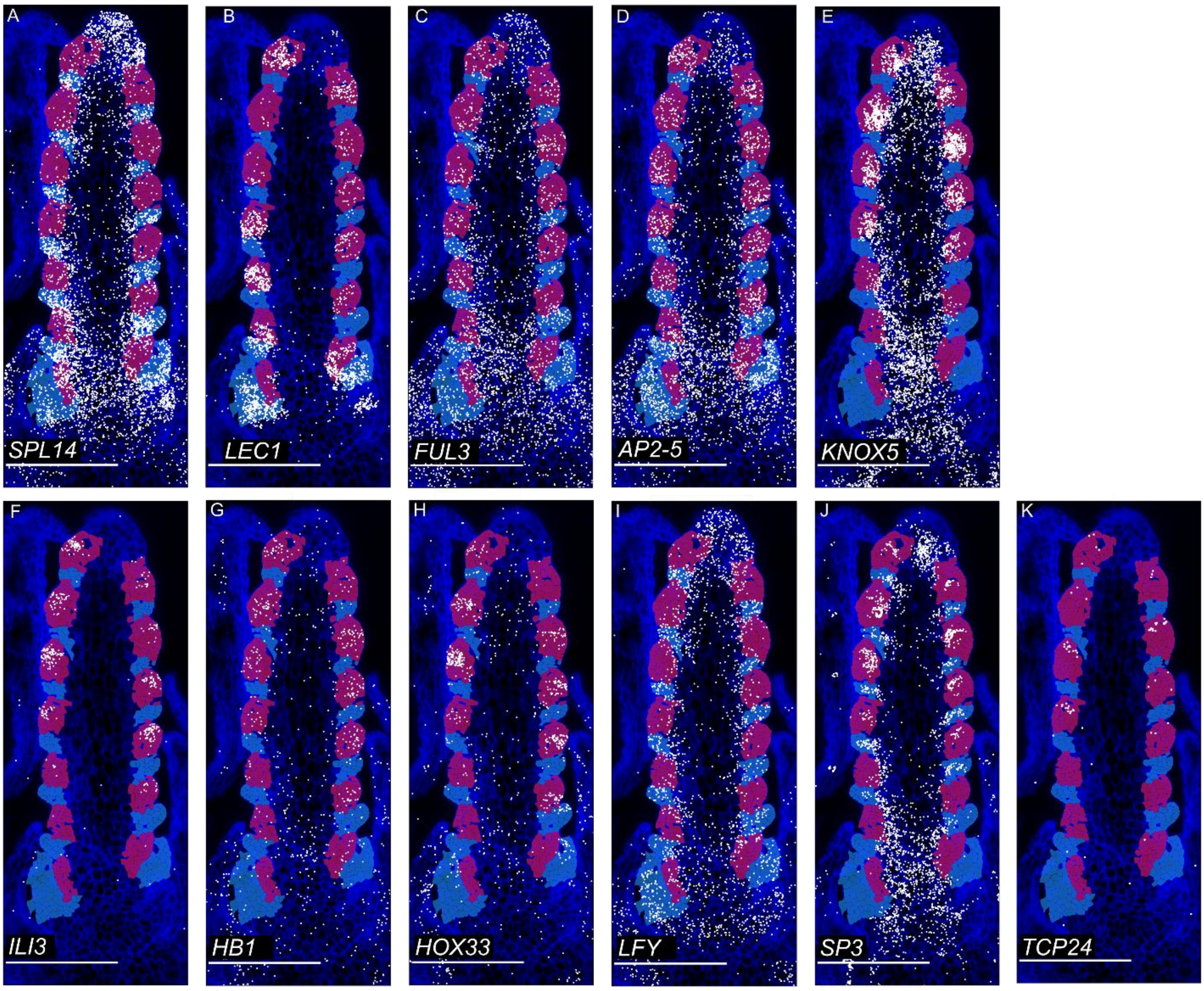

Supplementary Figure S11. Transcript Localisation of select genes in W2.5 inflorescence (*P1*^{WT}). **(A-J)** Transcript location of genes **(A)** *SQUAMOSA-PROMOTER BINDING PROTEIN-LIKE 14* (*SPL14*), **(B)** *LEAFY COTYLEDON1* (*LEC1*), **(C)** *FRUITFULL 3* (*FUL3*), **(D)** *AP2-LIKE 5* (*AP2-5*), **(E)** *KNOTTED1-LIKE HOMEOBOX 5* (*KNOX5*), **(F)** *INCREASED LEAF INCLINATION 3* (*ILI3*), **(G)** *HOMEOBOX DOMAIN 1* (*HB1*), **(H)** *HOMEOBOX 33* (*HOX33*), **(I)** *LEAFY* (*LFY*), **(J)** *SHORT PANICLE 3* (*SP3*), **(K)** *TCP24***.** Cells in each leaf ridge (LR) highlighted in blue and in each axillary meristem (AM) is highlighted in pink. Supports Figure 5.

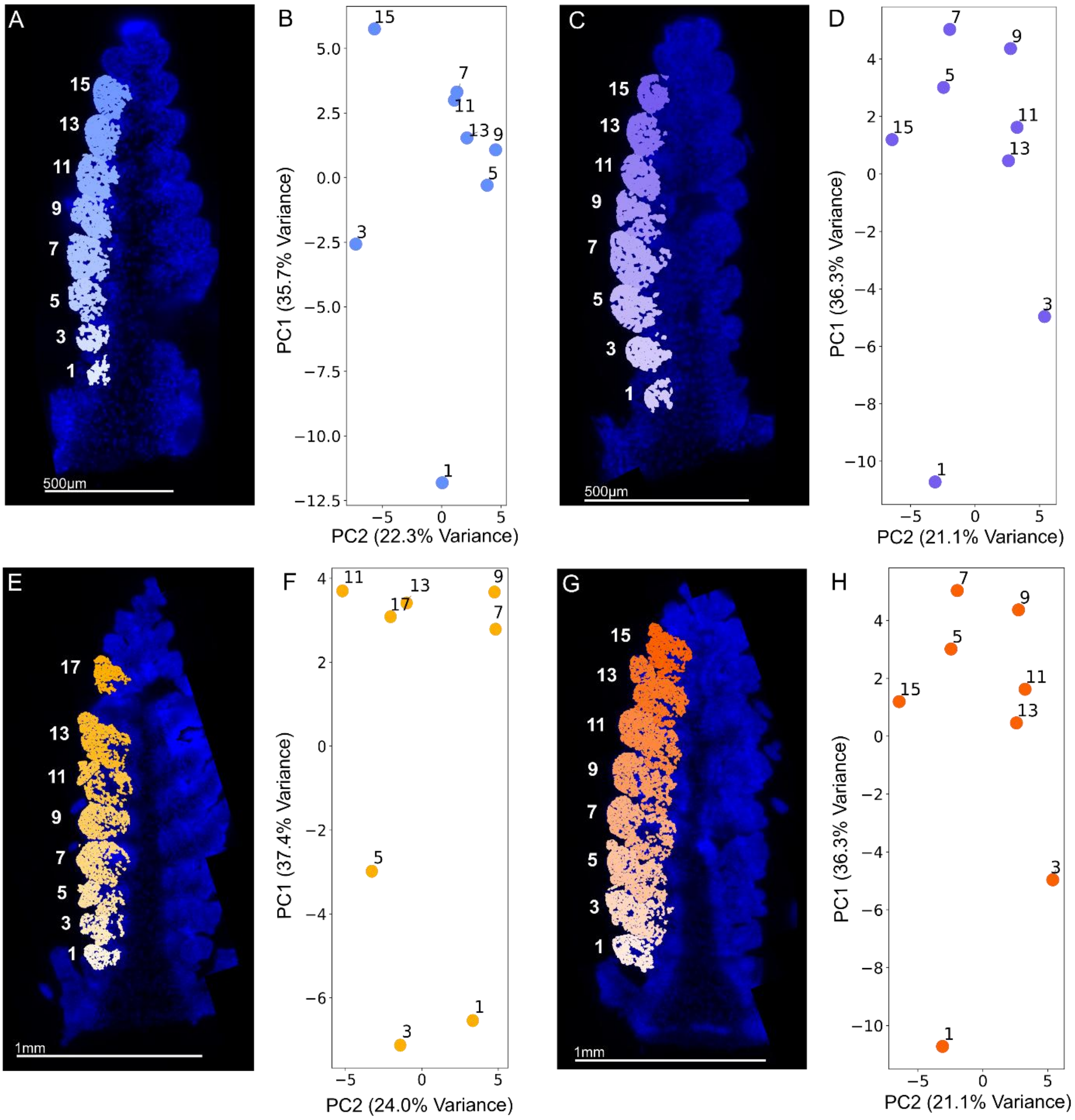

Supplementary Figure S12. Basal spikelets display distinct transcriptional signatures at stages W3.25 and W4. Spatial maps show the selection of cells in each spikelet along the apical–basal axis, with "1" representing the most basal spikelet. Principal component analysis was performed using a matrix of average transcript counts per cell from the 200-inflorescence gene panel in each spikelet group. Data are shown for **(A-B)** W3.25 *P1*^{WT} **(C-D)** W3.25 *P1*^{POL} **(E-F)** W4 *P1*^{WT} **(G-H)** W4 *P1*^{POL}. Basal spikelets are separated from central and apical regions in PC space, highlighting their distinct transcriptional programs. Supports Figure 5.
